# Big 5 Personality Traits and Individual- and Practice-Related Characteristics as Influencing Factors of Digital Maturity in General Practices: Quantitative Web-Based Survey Study

**DOI:** 10.2196/52085

**Published:** 2024-01-22

**Authors:** Lisa Weik, Leonard Fehring, Achim Mortsiefer, Sven Meister

**Affiliations:** 1 Health Care Informatics Faculty of Health, School of Medicine Witten/Herdecke University Witten Germany; 2 Helios University Hospital Wuppertal Department of Gastroenterology Witten/Herdecke University Wuppertal Germany; 3 Faculty of Health School of Medicine Witten/Herdecke University Witten Germany; 4 General Practice II and Patient-Centredness in Primary Care, Institute of General Practice and Primary Care Faculty of Health, School of Medicine Witten/Herdecke University Witten Germany; 5 Department Healthcare Fraunhofer Institute for Software and Systems Engineering ISST Dortmund Germany

**Keywords:** digital health, eHealth, digital maturity, maturity assessment, general practitioners, primary care physicians, primary care, family medicine, personality, digital affinity, digital health adoption

## Abstract

**Background:**

Various studies propose the significance of digital maturity in ensuring effective patient care and enabling improved health outcomes, a successful digital transformation, and optimized service delivery. Although previous research has centered around inpatient health care settings, research on digital maturity in general practices is still in its infancy.

**Objective:**

As general practitioners (GPs) are the first point of contact for most patients, we aimed to shed light on the pivotal role of GPs’ inherent characteristics, especially their personality, in the digital maturity of general practices.

**Methods:**

In the first step, we applied a sequential mixed methods approach involving a literature review and expert interviews with GPs to construct the digital maturity scale used in this study. Next, we designed a web-based survey to assess digital maturity on a 5-point Likert-type scale and analyze the relationship with relevant inherent characteristics using ANOVAs and regression analysis.

**Results:**

Our web-based survey with 219 GPs revealed that digital maturity was overall moderate (mean 3.31, SD 0.64) and substantially associated with several characteristics inherent to the GP. We found differences in overall digital maturity based on GPs’ gender, the expected future use of digital health solutions, the perceived digital affinity of medical assistants, GPs’ level of digital affinity, and GPs’ level of extraversion and neuroticism. In a regression model, a higher expected future use, a higher perceived digital affinity of medical assistants, a higher digital affinity of GPs, and lower neuroticism were substantial predictors of overall digital maturity.

**Conclusions:**

Our study highlights the impact of GPs’ inherent characteristics, especially their personality, on the digital maturity of general practices. By identifying these inherent influencing factors, our findings support targeted approaches to drive digital maturity in general practice settings.

## Introduction

### Background

Over the past decade, digital health solutions have garnered increasing attention owing to their transformative impact on health care systems worldwide [1]. Today, these have emerged as powerful tools in health care, revolutionizing the market by improving health outcomes [2,3], reducing costs [4], and improving the effectiveness and efficiency of health care delivery [4-6]. General practitioners (GPs) can choose various digital health solutions for their practice, ranging from remote monitoring of physiological parameters [7], video consultations [8], and mobile health apps [9] to digital appointment booking [10]. Thus, GPs must decide more than ever on their digital health agenda to successfully navigate the ever-changing landscape of digital health solutions and improve the quality of care [11].

To strategically develop a digital health agenda, it is crucial to evaluate the status quo [12]. Digital maturity assessment is a promising approach that allows evaluating health care facilities’ digital status across various technological and organizational dimensions to move toward an enhanced maturity stage [13,14]. As such, digital maturity is not a stable construct but should be considered as evolving toward a theoretical end point of maturity within the current digital health landscape, describing a successful development path toward digitalization along several stages. It is not a unidimensional construct but encompasses various technological and organizational dimensions beyond digital health adoption [13]. In essence, digital maturity provides a broader and more holistic perspective compared with digital health adoption, considering not only the adoption of specific digital tools but also an organization’s overall preparedness and capability to navigate the digital landscape. Accordingly, digital maturity can be considered a prerequisite to ensuring a successful digital transformation and optimized service delivery [12]. Although extensive research has studied digital maturity, its underlying dimensions [13-17], influencing factors [18], and associated outcomes [3,19] in inpatient care settings, only a few studies have undertaken similar investigations in general practice settings [20,21].

However, assessing digital maturity and its influencing factors is important in general practice settings. In European health care systems, GPs have a unique and central role in providing comprehensive and continuous health care services [22], as they are most patients’ first point of contact with the health care system [23]. This pivotal position places GPs at the forefront of digital transformation. Thus, the integration of digital technologies in general practices holds the promise of positively impacting a vast number of patients. Although the benefits of digital technologies in health care are evident [1], the full realization of these advantages hinges upon the level of digital maturity achieved by health care providers such as GPs.

### Objectives

Therefore, understanding the factors that drive digital maturity among GPs is vital for optimizing health care service delivery and harnessing the full potential of digital health solutions. To date, only a few studies have adopted a GP-focused approach and analyzed the influence of GP-related characteristics on digital maturity. Of these, the focus was on demographics and practice- and digital health use–related characteristics [21]. Although studies link technology adoption to personality [24-26], no study has investigated its influence on digital maturity more broadly. However, as GPs play a pivotal role in the digital maturity of their practices, we believe that analyzing the impact of their personality enables a comprehensive understanding of GP-related drivers of digital maturity, the development of targeted and effective measures to support GPs in their digitalization efforts, and the development of a dedicated and comprehensive maturity model for general practice settings. Thus, the objective of this study is to extend previous literature on influencing factors of digital maturity by shedding light on the role of GPs’ inherent characteristics in the digital maturity of general practices. By inherent characteristics, we mean those qualities that describe them personally and their practice. This study specifically focused on demographics, practice-related characteristics, digital health use, digital affinity, and personality. We aimed to evaluate the relationship between these inherent characteristics and the digital maturity of general practices.

## Methods

### Study Design

This study used a sequential approach involving qualitative and quantitative data collection and analysis. First, we conducted a literature review following the PRISMA-ScR (Preferred Reporting Items for Systematic Reviews and Meta-Analyses extension for Scoping Reviews) guideline [27] to identify relevant constituting dimensions and indicators of digital maturity across health care settings. We then conducted expert interviews with GPs following the COREQ (Consolidated Criteria for Reporting Qualitative Research) checklist [28] to validate and extend our literature review results, ensuring the relevance of the extracted indicators in general practice settings. In the next step, we designed a web-based survey following the CHERRIES (Checklist for Reporting Results of Internet E-Surveys) guideline [29] for internet surveys to assess digital maturity and relevant characteristics inherent to the GP and ultimately answer the following research question core to this study: Which demographic and practice-related characteristics, use-related variables, and personality traits substantially influence overall digital maturity?

### Ethical Considerations

All research project steps were approved by the Ethics Committee of Witten/Herdecke University (number S-242/2022). GPs willing to participate in the expert interviews or the web-based survey were required to provide informed consent before participation. They did not receive any incentive for participation. All data generated during the interviews were factually anonymized, meaning the data were presented and analyzed in aggregated or enlarged forms, which prevents conclusions from being drawn about individuals or only with disproportionate effort. Furthermore, all personal data collected in our web-based survey were anonymized by applying the k-anonymity principle, so that responses were deleted if they did not allow our anonymity criterion to apply.

### Literature Review

Our literature review followed the PRISMA-ScR guideline [27] (refer to Multimedia Appendix 1 for the completed PRISMA-ScR checklist). We searched the PubMed and PsycINFO databases for our literature review to gather relevant citations. As we aimed to identify and synthesize relevant indicators of digital maturity that could be adapted to general practice settings, we developed our search terms following other literature reviews (scoping and systematic) on digital maturity in the digital health context [13,16,17,30]. Thus, our search term included common variants of *digital maturity* combined using the Boolean OR operator (details on search terms are provided in Multimedia Appendix 1, item 8).

For our abstract and full-text screening, we excluded articles if they (1) were not related to digital health; (2) were not focused on digital maturity; (3) did not report on indicators, frameworks, or assessments of digital maturity, as extracting indicators was the main focus of our literature review; (4) were focused on national health care systems or inpatient care delivery, as these contexts essentially differ from general practice settings; (5) were focused on low-income countries and were thus not comparable to the German health care context; and (6) were not original, peer-reviewed, and published full-text articles. We decided not to limit our literature review to GPs because we aimed to validate the extracted indicators in qualitative expert interviews with GPs. Evidence from the included studies was synthesized by extracting and grouping potentially relevant indicators of digital maturity in line with the framework proposed in a recent review [13].

### Expert Interviews

As only limited research on digital maturity in general practice settings has been conducted to date [20], we conducted expert interviews with GPs to validate the indicators and dimensions identified in our literature review, ensure their relevance for general practices, and identify additional indicators relevant to digital maturity in general practices. The expert interviews followed the COREQ checklist for qualitative research [28] (refer to Multimedia Appendix 2 for the completed COREQ checklist). On the basis of the results of our literature review, we developed a semistructured interview guide to capture GPs’ perspectives on relevant indicators of digital maturity in general practices and their evaluation of the relevance of the proposed dimensions (refer to Multimedia Appendix 3 for the translated interview guide). The questions were designed to explore four main topics: (1) experience with digital health solutions, (2) perspectives on indicators of digital maturity, (3) perceived barriers toward the adoption and use of digital health solutions, and (4) preferable strategies to improve digital health adoption. This study focuses on the first 2 topics, whereas the latter will be part of subsequent analyses published elsewhere.

We recruited participants via email. The first interviewees were 1 colleague and 4 personal contacts of the authors. Next, the participants were purposively sampled via their publicly available email addresses to represent GPs from different regions and age groups. The interviewees were neither acquainted with each other nor were they in direct contact. Before the interview, participants received information about the overarching research design, the research question, and a broad description of the topics to be covered in the interview. They were also asked to provide informed consent. We then conducted the interviews in an internet-based one-on-one setting, video recorded them, and transcribed them verbatim to allow for subsequent qualitative analysis.

Data saturation was achieved after 10 interviews with the GPs. On average, the interviews lasted 45 minutes. Interview transcripts were then coded and analyzed using VERBI GmbH MAXQDA 2022 [31]. For our content analysis [32], we derived our coding scheme deductively based on the literature review results to allow for subsequent comparison. In addition, we derived themes inductively from the interview material, if mentioned by >1 interviewee, to provide additional insights. On the basis of this, we extracted a numeric score representing the number of interviews in which specific indicators of digital maturity were mentioned. These results were then compared with the results of our literature review to develop items for assessing digital maturity as part of our web-based survey.

### Web-Based Survey

#### Survey Design

The final and core part of our study was a cross-sectional survey among a convenience sample of GPs in Germany, investigating inherent characteristics and their digital maturity. The survey followed the CHERRIES checklist for internet surveys [29] (refer to Multimedia Appendix 4 for the completed CHERRIES; refer to Multimedia Appendix 5 for the survey questionnaire). Overall, the survey consisted of six sections: (1) demographics, practice-related characteristics, and digital health use; (2) GPs’ digital affinity; (3) big 5 personality traits; (4) digital maturity of the practice; (5) perceived barriers to digital health adoption and use; and (6) potential measures to support digital health adoption. This paper discusses only the results of sections 1 to 4, as our goal was to explore the association between overall digital maturity and personality characteristics. An introductory page informed the participants about the research purpose, goals, target population, length of the survey, and institutional review board approval. On the next page, we informed the participants about the data security and storage policies and the researchers involved. The participants had to provide informed consent to continue with the survey.

#### Measures

To capture the participants’ demographics and practice-related characteristics, we used single-choice questions concerning their gender, age range, practice location, professional experience, patient population, and type of practice. We further assessed digital health use (current use and expected future use of digital health solutions) and the perceived digital affinity of medical assistants using 5-point Likert-type scales.

We used the German version of the Affinity for Technology Interaction scale [33] to capture GPs’ digital affinity. The 9-item scale captures a person’s tendency to actively engage in intensive technology interaction on a 6-point Likert-type scale.

We assessed the GPs’ personality using a German-language scale [34] (Big Five Inventory–Short Version). The 21-item scale assesses the big 5 personality traits of extraversion, agreeableness, conscientiousness, neuroticism, and openness on a 5-point Likert-type scale.

The 28 items for our digital maturity assessment were developed based on the results of our literature review and expert interviews. We used items for all digital maturity indicators proposed by >3 theoretical maturity models in our literature review or mentioned by >1 interviewee in our expert interviews to ensure theoretical and expert consensus. For all other indicators, we consulted an expert in the field of digital maturity before excluding them from the survey to ensure that all key indicators were covered. The items were then developed based on the theoretical descriptions of the corresponding digital maturity indicators identified in our literature review. Although some indicators could be covered in their entirety by 1 item (eg, *Change Management*), others were more complex in nature, thus requiring more items to cover them fully (eg, the indicator *Systems and Services* was captured by 5 items to cover various digital health solutions). All items were discussed and iteratively refined between the authors. In total, the items covered 7 constituting dimensions of digital maturity: *Governance and Management*, *IT Capability*, *People, Skills and Behavior*, *Interoperability*, *Strategy*, *Data Analytics*, and *Patient-centered Care*. Participants were asked to rate their agreement with the items on a 5-point Likert-type scale.

#### Pretest and Recruitment

To test the final questionnaire for clarity, comprehensiveness, usability, and technical functionality, we conducted a pretest with 15 physicians working in ambulatory care settings. On the basis of the pretest, we refined our welcome message and the wording of some items. The survey was then conducted between April and August 2023. We disseminated the final survey via various recruitment channels. These included interview participants and personal contacts of the researchers, teaching practices of affiliated universities, social networks such as LinkedIn, physician networks, research practice networks, and mailing lists. Personal contacts and social networks were addressed directly by the authors. For teaching practices, physician networks, research practice networks, and mailing lists, we initially contacted the respective person responsible via mail. The person responsible then disseminated the survey access link within the network via their communication channels and announcements. The survey was conducted in an open-access mode, meaning anyone with an access link could participate. In addition, we did not track which invited participants had started or completed the survey, thus limiting our ability to use reminders. We also did not provide incentives to participants. The final survey took approximately 10 to 15 minutes to complete.

#### Data Cleaning and Analysis

**Figure 1:**
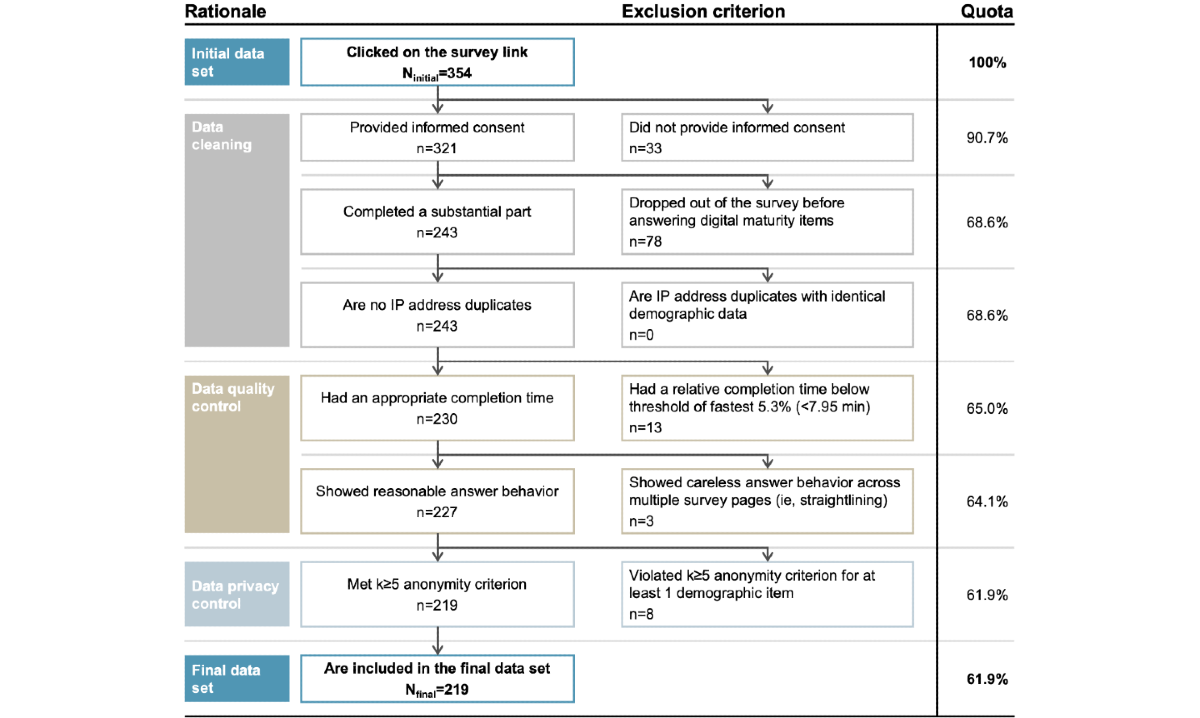
Overview of the data cleaning approach. Straightlining was monitored as part of our data quality control procedure, excluding respondents who showed a straight line across >2 survey pages.

Before our statistical analysis, we performed thorough cleaning of the data obtained. Following common procedures [35], our data cleaning included the removal of responses without informed consent, incomplete responses, and duplicate entries, leading to 111 of the initial 354 responses (3.1%) being excluded. In addition, we applied data quality control procedures in line with practical recommendations [36]. Therefore, we excluded responses with very low completion times below our threshold of the fastest 5.3% (12/243) of respondents. In addition, we excluded respondents who showed careless answer behavior across multiple survey pages and thus in >1 item battery, that is, who chose the very same answer option for all items in >1 item battery, as this might indicate careless responding as opposed to straightlining owing to respondents’ actual views [36]. We further removed all responses violating our k≥5 anonymity criterion to comply with data privacy regulations as part of the institutional review board approval. Consequently, we removed all questionnaires with <5 respondents per demographic item answer option. Ultimately, from the 354 participants who initially clicked on the survey link, responses from 219 (61.9%) respondents were included in our analysis (Figure 1).

We first computed the mean value for respondents’ digital affinity, personality traits, and digital maturity dimensions to analyze the association between digital maturity and GPs’ inherent characteristics. As we focused our statistical analysis on digital maturity more broadly instead of individual dimensions, we additionally computed an overall digital maturity score as the average score across all items. All subsequent statistical analyses were conducted with SPSS version 29.0 (IBM Corp) for Macintosh [37].

For all scales, Cronbach α [38] was used to assess the internal consistency of the scales. An overview can be found in Table 1. The internal consistency (Cronbach α=.92) for the Affinity for Technology Interaction scale is in line with previous research [33] and can be considered excellent. Internal consistencies for the big 5 personality traits can be regarded as acceptable or good. In comparison with previous literature [34], the internal consistencies in our sample differed. In particular, the internal consistency for conscientiousness was lower than that in the original study [34]. The overall high conscientiousness and low variability of the score in this sample might explain this. GPs constitute a relatively homogeneous sample for conscientiousness, which tends to lower the Cronbach α estimates. As we derived our digital maturity scale based on our literature review and qualitative interviews, no comparison with previous literature is possible. Internal consistencies can be considered acceptable for all subscales with >2 items. Only the Interoperability subscale had a Cronbach α that was well below the .70 threshold, but as this subscale consists of only 2 items, an interpretation of the internal consistency cannot be made [38].

**Table 1:** Sample size, mean, SD, number of items, and internal consistencies of the scales used.

Scale			Values, n (%)		Values, mean (SD)		Items, n		Cronbach α (original)		Cronbach α (current)
Digital affinity (ATI^a^ scale)			219 (100)		3.64 (1.20)		9		.89		.92
**Personality (BFI-K^b^ scale)**											
	Extraversion	219 (100)		3.62 (0.80)		4		.86		.81	
	Agreeableness	219 (100)		3.53 (0.76)		4		.64		.68	
	Conscientiousness	219 (100)		4.10 (0.58)		4		.70		.63	
	Neuroticism	219 (100)		2.42 (0.70)		4		.74		.71	
	Openness	219 (100)		3.81 (0.69)		5		.66		.74	
**Digital maturity**											
	Governance and management	218 (99.5)		3.97 (0.74)		6		—^c^		.87	
	IT capability	219 (100)		2.76 (0.73)		9		—^c^		.70	
	People, skills and behavior	219 (100)		4.00 (0.63)		4		—^c^		.71	
	Interoperability	219 (100)		3.60 (0.92)		2		—^c^		.54^d^	
	Strategy	219 (100)		3.51 (0.94)		2		—^c^		.69^d^	
	Data analytics	219 (100)		3.17 (1.22)		2		—^c^		.89^d^	
	Patient-centered care	219 (100)		2.18 (0.94)		3		—^c^		.71	

^a^ATI: Affinity for Technology Interaction.

^b^BFI-K: Big Five Inventory–Short Version.

^c^All digital maturity subscales were developed based on the literature review and expert interviews. Thus, Cronbach α for the original studies cannot be shown.

^d^As the subscales *Interoperability*, *Strategy*, and *Data Analytics* only comprise 2 items, an interpretation of their internal consistencies should not be made.

We conducted separate ANOVAs with 2-tailed significance (*P*<.05) to assess differences in overall digital maturity, given the several inherent characteristics relevant to our study. For some of our independent variables (GPs’ inherent characteristics), Q-Q plots and Shapiro-Wilk test showed that the overall digital maturity score did not follow a normal distribution for all levels. Levene test further showed heteroscedasticity in some instances. Following practical recommendations [39], we used Welch *F* [40] for all ANOVAs to adjust for the heteroscedasticity in the mentioned instances. Where ANOVAs revealed a significant difference (*P*<.05) in overall digital maturity, we investigated post hoc procedures. As sample sizes vary across groups, we used Hochberg GT2 (homogeneity of variance met) or Games-Howell (homogeneity of variance not met) as post hoc procedures[39]. Although we aimed to assess differences in overall digital maturity based on digital affinity and personality, we additionally clustered participants into 3 categories (low, moderate, and high) for each variable based on the underlying Likert-type scales. Thus, participants scoring in the bottom third of the Likert-type scale were categorized as low, those in the second third as moderate, and those in the upper third as high. As this categorization does not cover the whole spectrum of the continuous underlying variable, it was only used in our ANOVAs as an initial indicator for differences in these variables between GPs. These differences were then analyzed more specifically in a linear regression model using the continuous variables without categorization.

We conducted a linear regression analysis to deepen our understanding of the relationship between overall digital maturity and the independent variables. We followed a hierarchical approach for entering variables into our model to determine the influence of our demographic and practice-related variables on overall digital maturity and to separate this from the influence of digital health use, digital affinity, and personality. All nominal or ordinal variables—age, practice location size, professional experience, practice type, and current use—were dummy coded before being entered into the model. Before entering the predictors into the model, we assessed their potential multicollinearity using variance inflation factor and tolerance values following practical recommendations [39]. As all variance inflation factor values were <10 and tolerance values were >0.1, multicollinearity does not seem to flaw our analysis. In the first stage of our approach, we only included demographics and practice-related characteristics, that is, gender, age, practice location size, professional experience, and practice type. The second stage additionally included variables related to digital health use, that is, the current use and the expected future use. In model 3, we added digital affinity–related variables, that is, the perceived digital affinity of medical assistants and GPs. For the final model, we also included personality traits, that is, extraversion, agreeableness, conscientiousness, neuroticism, and openness. We chose the described sequence of insertion based on prior research and theoretical reasoning, with variables that were analyzed in previous research entered earlier into the model and the blocks, each covering different categories of inherent variables, that is, demographics and practice-related characteristics, digital health use, digital affinity, and personality.

## Results

### Characterizing Digital Maturity in General Practices (Literature Review and Expert Interview Results)

**Figure 2:**
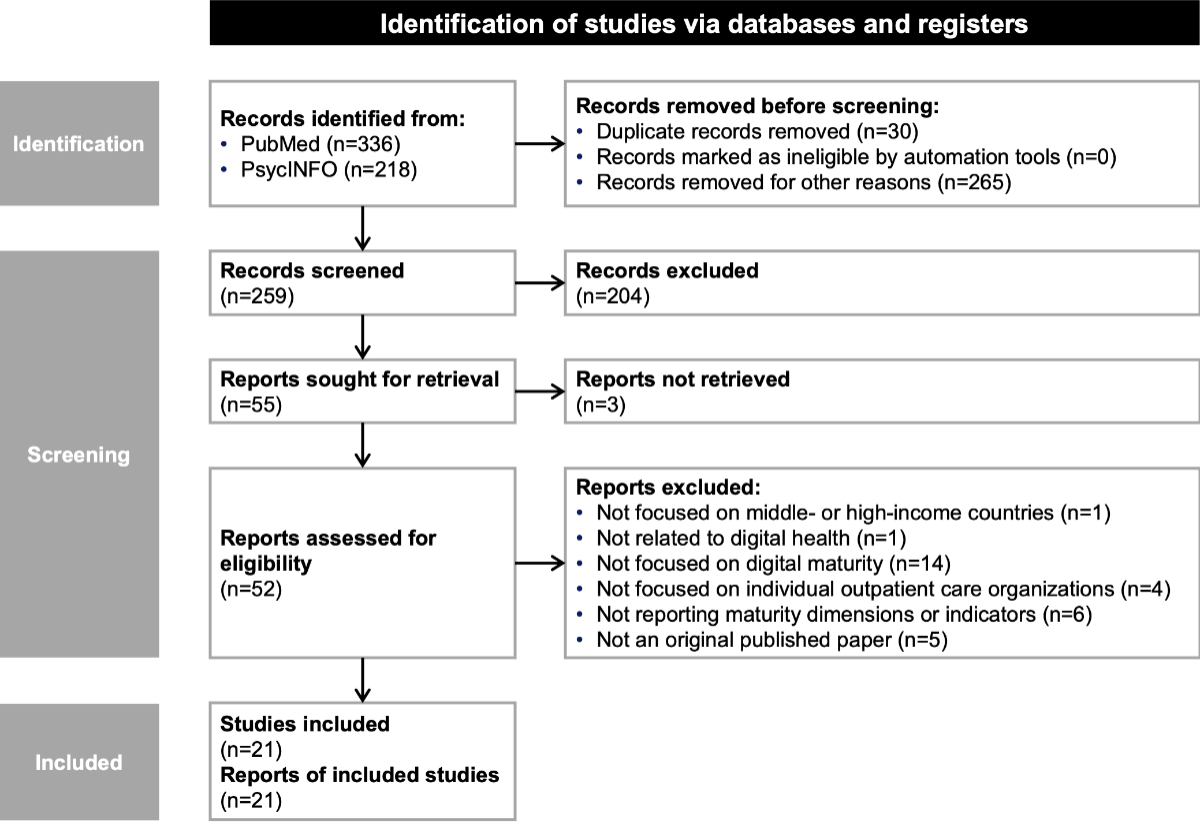
Flowchart for the literature review following PRISMA-ScR (Preferred Reporting Items for Systematic Reviews and Meta-Analyses extension for Scoping Reviews) guidelines. Records removed for other reasons shows records removed based on our language and publication date criteria.

We narrowed the 554 initially identified citations to studies published in English or German between January 2018 and December 2022 to account for more recent research findings. For these, we conducted abstract screening based on the predefined inclusion criteria, leading to 55 potentially relevant articles being retained. Next, we performed a full-text review to determine eligibility, resulting in 21 papers [3,13-16,41-56] being included after the screening. A detailed overview of the screening process is shown in Figure 2.

A total of 17 distinct maturity models were presented and used in the studies, with only around half looking at people- and skill-related indicators of digital maturity [13,16,43,44,46-48,50,53,56] (n=10, 58%). Of these, technology use (8/10, 80%) was the most cited indicator of digital maturity [13,16,43,47,48,50,53,56]. All other indicators in this dimension were only present in 3 (18%) of the 17 maturity models, covering the aspects of education and training [13,43,44], knowledge management [13,43,46], and individual competence [13,43,47]. The literature review identified the following 7 constituting dimensions of digital maturity: *Governance and Management*, *IT Capability*, *People, Skills and Behavior*, *Interoperability*, *Strategy*, *Data Analytics*, and *Patient-centered Care*.

**Figure 3:**
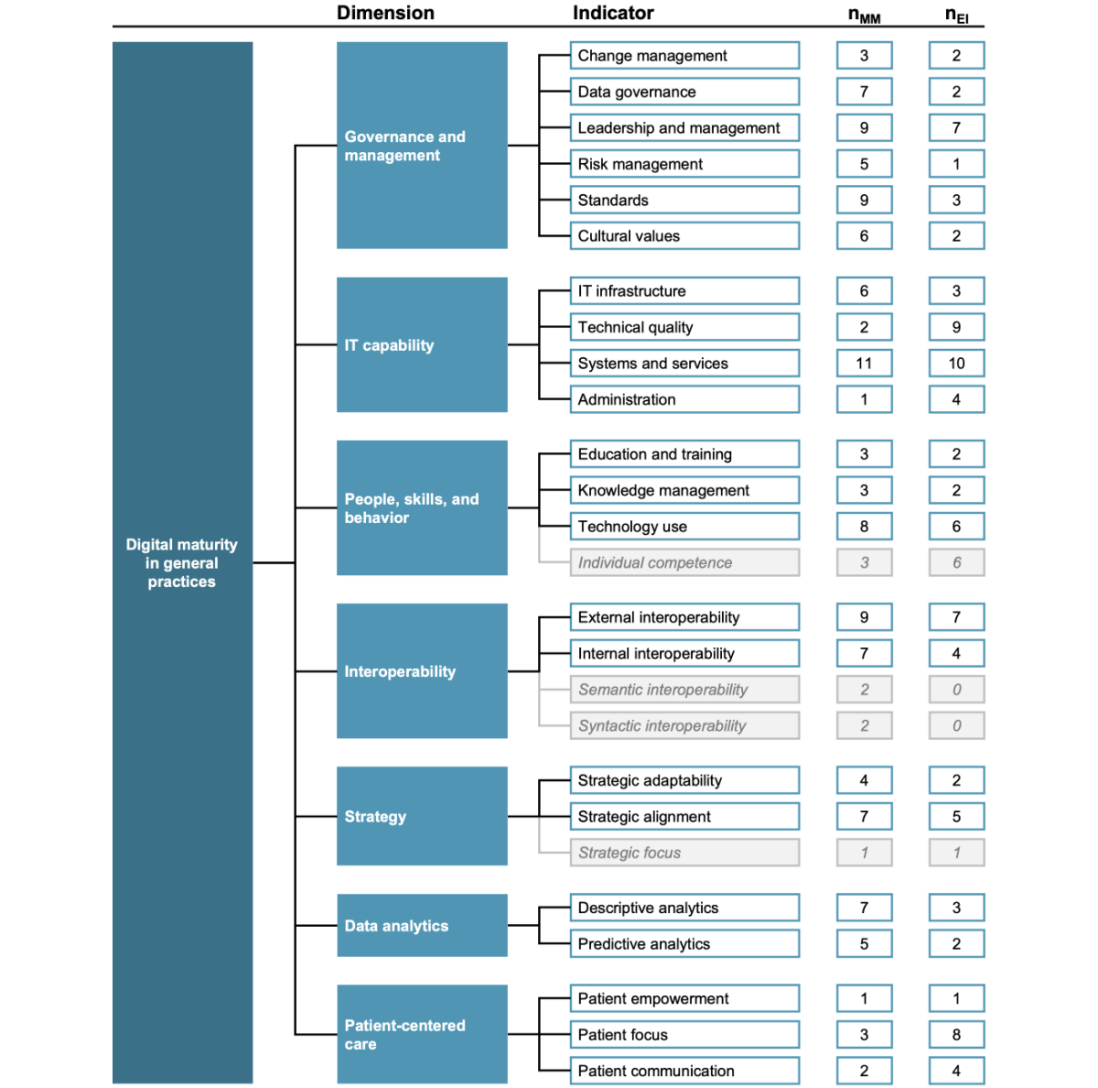
Overview of the constituting digital maturity dimensions and indicators based on the literature review and expert interview results. nMM represents the number of maturity models proposing the respective indicator; nEI shows the number of expert interviews in which the indicator was mentioned. All indicators proposed in >3 maturity models or mentioned in >1 interview were included in the survey. Light grey boxes with italic text show indicators not included in the subsequent web-based survey. Individual competence was not included as we assessed general practitioners’ digital affinity separately; Patient empowerment was included owing to expert consensus.

Participants in our expert interviews were aged between 32 and 68 (mean 53.1, SD 12.7) years and worked as GPs for 1 to 36 (mean 17.7, SD 12.1) years in cities with approximately 4000 to 600,000 inhabitants. Four GPs worked in a single practice, 5 in a group practice, and 1 in a medical care center. The results of our expert interviews validated the relevance of all 7 constituting dimensions in general practice settings. The interviews revealed that GPs especially perceived the availability of digital systems and tools and their quality as relevant indicators of digital maturity. In line with a high estimated relevance (8.6/10.0), people- and skill-related indicators were mentioned by almost all interviewees (9/10, 90%). The dimension of *Patient-centered Care* was rated as least relevant concerning the digital maturity of practices (6.5/10.0). In our subsequent web-based survey, we included items for 22 digital maturity indicators based on the aforementioned inclusion criteria. The *Patient Empowerment* indicator was included after expert consensus, as it was considered a core value proposition of digital health solutions. An overview of the included digital maturity dimensions and indicators based on the synthesized results of our literature review and expert interviews is presented in Figure 3.

### Exploring Influencing Factors of Overall Digital Maturity in General Practices (Web-Based Survey Results)

As we aimed to analyze potentially relevant influencing factors inherent to GPs, our web-based survey specifically assessed four areas of inherent characteristics: (1) demographics and practice-related characteristics, (2) digital health use, (3) digital affinity, and (4) personality.

#### GPs’ Inherent Characteristics and Digital Maturity

Demographics, practice-related characteristics, and the digital health use of the participating GPs are summarized in Table 2. After data cleaning, quality control, and privacy control, the final sample consisted of 219 GPs, covering approximately 0.4% (219/55,112) of the German GP population [57]. The respondents were predominantly women (122/219, 55.7%), with a median age of 46 to 55 years. Approximately half of the respondents worked in relatively small town areas, either in towns with 5000 to 20,000 inhabitants (67/219, 30.6%) or in smaller villages with <5000 inhabitants (45/219, 20.5%).

**Table 2:** Characteristics of the participating general practitioners (N=219).

Characteristic and category		Values, n (%)
**Gender**		
	Man	97 (44.3)
	Woman	122 (55.7)
	Nonbinary	0 (0)
	No Answer	0 (0)
**Age (y)**		
	<26	0 (0)
	26-35	21 (9.6)
	36-45	60 (27.4)
	46-55	55 (25.1)
	56-65	73 (33.3)
	>65	10 (4.6)
**Practice location size (number of inhabitants)**		
	<5000	45 (20.5)
	5000-20,000	67 (30.6)
	20,001-100,000	44 (20.1)
	100,001-500,000	28 (12.8)
	>500,000	35 (16)
**Professional experience (y)**		
	<1	0 (0)
	1-5	20 (9.1)
	6-10	35 (16)
	11-20	59 (26.9)
	21-30	60 (27.4)
	>30	45 (20.5)
**Patient population**		
	Only privately health-insured	0 (0)
	Only statutory health-insured	0 (0)
	Both	219 (100)
**Practice type**		
	Single practice	102 (46.5)
	Practice sharing	12 (5.5)
	Group practice	93 (42.5)
	Practice clinic	0 (0)
	Practice network	0 (0)
	Medical care center	12 (5.5)
	Collaborative laboratory	0 (0)
**Current use of digital health solutions**		
	Never	18 (8.2)
	Less than once per month	48 (21)
	Monthly	24 (11)
	Weekly	32 (14.6)
	Daily	99 (45.2)
**Expected future use of digital health solutions**		
	Very unlikely	10 (4.6)
	Rather unlikely	30 (13.7)
	Neither unlikely nor likely	15 (6.8)
	Rather likely	48 (21.9)
	Very likely	116 (53)
**Perceived digital affinity of medical assistants**		
	Not at all digitally savvy	3 (1.4)
	Rather not digitally savvy	43 (19.6)
	Neither not digitally savvy nor digitally savvy	55 (25.1)
	Rather digitally savvy	93 (42.5)
	Very digitally savvy	25 (11.4)

Most respondents (164/219, 74.9%) had >10 years of professional experience . They predominantly worked in either single practices (102/219, 46.5%) or group practices (93/219, 42.5%). All respondents (219/219, 100%) treated privately and statutory health-insured patients .

Although approximately half of the respondents (99/219, 45.2%) used digital health solutions daily, almost one-third (64/219, 29.2%) did not use them at all or seldom. However, most respondents (116/219, 53%) were very likely to use digital health solutions in the future.

With regard to digital affinity, most respondents perceived their medical assistants to be moderately (55/219, 25.1%) or rather digitally savvy (93/219, 42.5%) and had a relatively moderate digital affinity [33] (mean 3.64, SD 1.20) themselves.

Regarding personality [34], on average, respondents showed high conscientiousness (mean 4.10, SD 0.58) and openness (mean 3.81, SD 0.69), moderate extraversion (mean 3.62, SD 0.80) and agreeableness (mean 3.53, SD 0.76), and low to moderate neuroticism (mean 2.42, SD 0.70).

**Figure 4:**
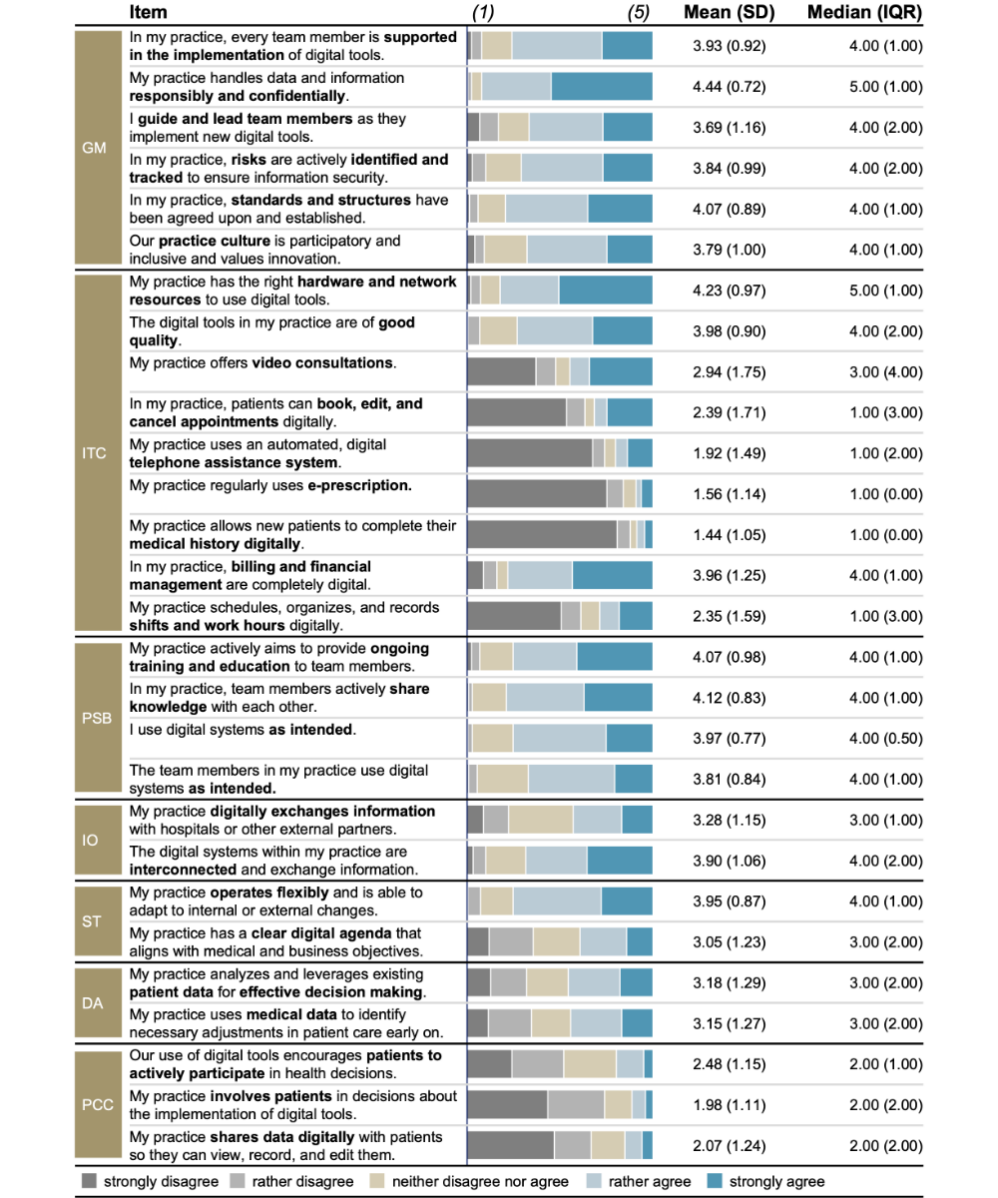
Frequencies, mean, and SD for digital maturity items along the constituting dimensions assessed (N=219). The bar chart shows the number of respondents per answer category. DA: data analytics; GM: governance and management; IO: interoperability; ITC: IT capability; PCC: patient-centered care; PSB: people, skills, and behavior; ST: strategy.

Our sample’s overall digital maturity was moderate (mean 3.31, SD 0.64) based on the 5-point Likert-type scale used. Among the 7 constituting dimensions analyzed in this study, *People, Skills, and Behavior* (mean 4.00, SD 0.63) and *Governance and Management* (mean 3.97, SD 0.74) registered the highest average scores, followed by moderate scores for *Interoperability* (mean 3.60, SD 0.92), *Strategy* (mean 3.51, SD 0.94), *Data Analytics* (mean 3.17, SD 1.22), and *IT Capability* (mean 2.76, SD 0.73). Interestingly, *Patient-centered Care* received the lowest average value (mean 2.18, SD 0.94). For most individual digital maturity items, scores were moderate to high, with the highest ratings for *data security and confidentiality* (mean 4.44, SD 0.72; median 5.00, IQR 1.00) and *hardware and network resources* (mean 4.24, SD 0.97; median 5.00, IQR 1.00). A detailed overview of the individual item scores is provided in Figure 4.

#### Comparing Differences in Overall Digital Maturity Based on GPs’ Inherent Characteristics (ANOVA Results)

We found significant omnibus differences when comparing overall digital maturity scores based on the different areas of GPs’ inherent characteristics (refer to Table 3 for ANOVA results and Table 4 for post hoc test results).

**Table 3:** ANOVAs for overall digital maturity (N=219).

Variable			Welch *F* (*df*_*1*_*, df*_*2*_)		*P* value
**Demographics and practice-related characteristics**					
	Gender^a^	2.362 (217)		.01	
	Age	1.341 (4,46.40)		.27	
	Practice location size	0.256 (4,94.10)		.91	
	Professional experience	1.557 (4,80.48)		.19	
	Practice type	0.511 (3,29.31)		.68	
**Digital health use**					
	Current use	2.200 (4,65.41)		.08	
	Future use	2.843 (4,39.40)		.04	
**Digital affinity**					
	MAs’^b^ digital affinity	4.813 (4,14.39)		.01	
	GPs’^c^ level of digital affinity	8.504 (2,103.66)		.001	
**Personality**					
	Level of extraversion	4.753 (2,62.41)		.01	
	Level of agreeableness	0.087 (2,42.89)		.92	
	Level of conscientiousness	0.537 (2,2.63)		.64	
	Level of neuroticism	7.552 (2,30.04)		.002	
	Level of openness	0.073 (2,16.56)		.93	

^a^As gender is a dichotomous variable, we conducted a 2-tailed *t* test instead of an ANOVA, with the results showing the *t* statistic (in the column *Welch F*), df, and *P* value.

^b^MA: medical assistant.

^C^GP: general practitioner.

In our sample, overall digital maturity differed significantly between men and women (t_217_=2.362; *P*=.01), with men reporting a higher digital maturity (mean 3.42, SD 0.66; Cohen *d*=0.32). We did not find substantial differences in overall digital maturity for age, practice location size, professional experience, or practice types.

In addition, there was no substantial difference in overall digital maturity based on the current use of digital health solutions. We found significant differences in overall digital maturity based on the expected future use of digital health solutions (Welch *F*_4,39.40_=2.843; *P*=.04). Respondents who expected to very likely use digital health solutions in the future had a higher overall digital maturity (mean 3.45, SD 0.66) compared with respondents reporting a rather low likelihood of future use (mean 3.10, SD 0.70). However, this difference was not statistically significant (*P*=.07; Cohen *d*=−0.52).

Furthermore, we found significant differences in overall digital maturity based on both the perceived digital affinity of medical assistants (Welch *F*_4,14.39_=4.813; *P*=.01) and the level of respondents’ digital affinity (Welch *F*_2,103.66_=8.504; *P*=.001). Respondents who perceived their medical assistants to be rather digitally savvy (mean 3.37, SD 0.65; *P*=.01; Cohen *d*=−0.59) or very digitally savvy (mean 3.69, SD 0.70; *P*=.001; Cohen *d*=−1.12) had a significantly higher overall digital maturity compared with respondents who perceived their medical assistants to be somewhat not digitally savvy (mean 3.00, SD 0.56). In addition, the overall digital maturity was significantly higher for respondents with a high level of digital affinity (mean 3.57, SD 0.71) compared with those with moderate (mean 3.26, SD 0.54; *P*=.004; Cohen *d*=−0.50) or low levels (mean 3.07, SD 0.57; *P*=.001; Cohen *d*=−0.76).

Regarding personality, we found significant differences in overall digital maturity based on the level of extraversion (Welch *F*_2,62.41_=4.753; *P*=.01) and neuroticism (Welch *F*_2,30.04_=7.552; *P*=.002). Hochberg GT2 post hoc tests revealed that the overall digital maturity was significantly higher for respondents with high levels of extraversion (mean 3.43, SD 0.65) compared with respondents with moderate levels of extraversion (mean 3.17, SD 0.62; *P*=.01; Cohen *d*=−0.41). In addition, respondents with low levels of neuroticism had a significantly higher overall digital maturity (mean 3.45, SD 0.62) compared with those with moderate (mean 3.20, SD 0.60; *P*=.009; Cohen *d*=0.41) or high levels of neuroticism (mean 2.82, SD 0.70; *P*=.002; Cohen *d*=1.00).

**Table 4:** Post hoc tests for significant ANOVAs for digital maturity (N=219).

Variable and category		Values, n (%)	Values, mean (SD)	Group comparison	*P* value	Cohen *d*
**Gender**						
	Men	97 (44.3)	3.42 (0.66)	Vs women	.01	0.32
	Women	122 (55.7)	3.22 (0.61)	N/A^a^	N/A	N/A
**Future use**						
	Very unlikely	10 (4.6)	3.09 (0.68)	Vs rather unlikely	.99	−0.01
	Very unlikely	10 (4.6)	3.09 (0.68)	Vs neither unlikely nor likely	.99	−0.05
	Very unlikely	10 (4.6)	3.09 (0.68)	Vs rather likely	.99	−0.24
	Very unlikely	10 (4.6)	3.09 (0.68)	Vs very likely	.57	−0.54
	Rather unlikely	30 (13.7)	3.10 (0.70)	Vs neither unlikely nor likely	.99	−0.03
	Rather unlikely	30 (13.7)	3.10 (0.70)	Vs rather likely	.99	−0.21
	Rather unlikely	30 (13.7)	3.10 (0.70)	Vs very likely	.07	−0.52
	Neither unlikely nor likely	15 (6.8)	3.12 (0.54)	Vs rather likely	.99	−0.20
	Neither unlikely nor likely	15 (6.8)	3.12 (0.54)	Vs very likely	.45	−0.20
	Rather likely	48 (21.9)	3.22 (0.50)	Vs very likely	.33	−0.37
	Very likely	116 (53)	3.45 (0.66)	N/A	N/A	N/A
**MA^b^ digital affinity**						
	Not at all digitally savvy	3 (1.4)	2.72 (0.86)	Vs rather not digitally savvy	.99	−0.49
	Not at all digitally savvy	3 (1.4)	2.72 (0.86)	Vs neither not digitally savvy nor digitally savvy	.66	−1.14
	Not at all digitally savvy	3 (1.4)	2.72 (0.86)	Vs rather digitally savvy	.51	−0.99
	Not at all digitally savvy	3 (1.4)	2.72 (0.86)	Vs very digitally savvy	.09	−1.36
	Rather not digitally savvy	43 (19.6)	3.00 (0.56)	Vs neither not digitally savvy nor digitally savvy	.14	−0.59
	Rather not digitally savvy	43 (19.6)	3.00 (0.56)	Vs rather digitally savvy	.01	−0.59
	Rather not digitally savvy	43 (19.6)	3.00 (0.56)	Vs very digitally savvy	<.001	−1.12
	Neither not digitally savvy nor digitally savvy	55 (25.1)	3.31 (0.50)	Vs rather digitally savvy	1.00	−0.10
	Neither not digitally savvy nor digitally savvy	55 (25.1)	3.31 (0.50)	Vs very digitally savvy	.10	−0.67
	Rather digitally savvy	93 (42.5)	3.37 (0.65)	Vs very digitally savvy	.20	−0.48
	Very digitally savvy	25 (11.4)	3.69 (0.70)	N/A	N/A	N/A
**GPs’^c^ level of digital affinity**						
	Low digital affinity	44 (20.1)	3.07 (0.57)	Vs moderate digital affinity	.21	−0.33
	Low digital affinity	44 (20.1)	3.07 (0.57)	Vs high digital affinity	<.001	−0.76
	Moderate digital affinity	112 (51.1)	3.26 (0.57)	Vs high digital affinity	.004	−0.50
	High digital affinity	63 (28.8)	3.57 (0.71)	N/A	N/A	N/A
**Level of extraversion**						
	Low level of extraversion	21 (9.6)	3.17 (0.48)	Vs moderate level of extraversion	1.00	0.00
	Low level of extraversion	21 (9.6)	3.17 (0.48)	Vs high level of extraversion	.22	−0.41
	Moderate level of extraversion	82 (37.4)	3.17 (0.62)	Vs high level of extraversion	.01	−0.41
	High level of extraversion	116 (53)	3.43 (0.65)	N/A	N/A	N/A
**Level of neuroticism**						
	Low level of neuroticism	114 (52.1)	3.45 (0.62)	Vs moderate level of neuroticism	.009	0.41
	Low level of neuroticism	114 (52.1)	3.45 (0.62)	Vs high level of neuroticism	.002	1.00
	Moderate level of neuroticism	93 (42.5)	3.20 (0.60)	Vs high level of neuroticism	.13	0.62
	High level of neuroticism	12 (5.5)	2.82 (0.70)	N/A	N/A	N/A

^a^N/A: not applicable.

^b^MA: medical assistant.

^c^GP: general practitioner.

#### Exploring the Relationship Between Overall Digital Maturity and GPs’ Inherent Characteristics (Regression Model Results)

An overview of our regression model can be found in Table 5.

When only including demographics and practice-related characteristics, our model was not significant (*F*_16,202_=0.865; *P*=.61; *R^2^*=0.064). Including digital health use in our model yielded a significant improvement, but the model remained insignificant (*F*_21,197_=1.274; *P*=.20; *R^2^*=0.120; Δ *R^2^* =0.056; Δ*P*=.03). However, when including digital affinity–related variables in stage 3, our model reached statistical significance (*F*_23,195_=2.676; *P*=.001). Adding these variables to the model significantly increased the proportion of the explained criterion variance to 24% (Δ*P*=.001). Including personality-related variables in our final model led to a significant increase in *R^2^* of approximately 7% (Δ*P*=.003) to an overall *R^2^* of 31% (*F*_28,190_=3.029; *P*=.001).

**Figure 5:**
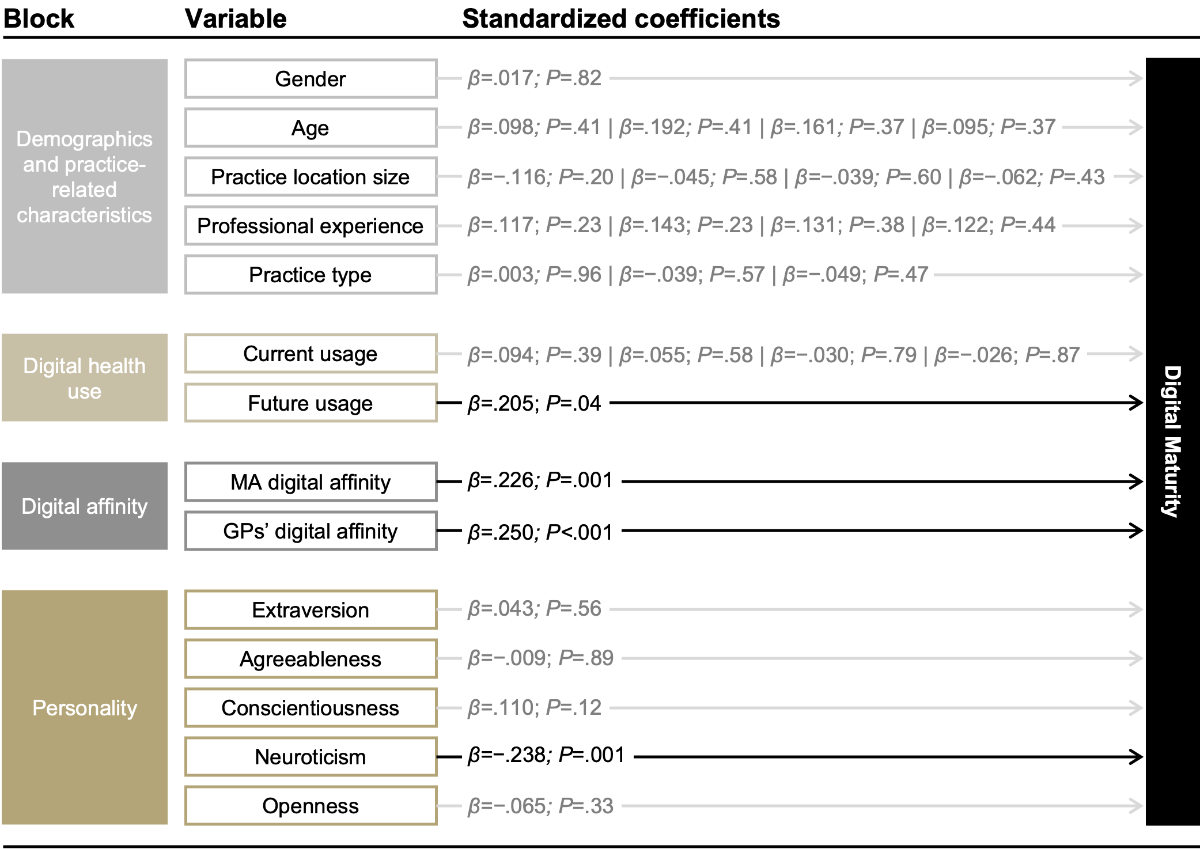
Standardized coefficients for the final regression model predicting overall digital maturity (N=219). Age, practice location size, professional experience, practice type, and current use were dummy coded for the analysis. Age: 36-45, 46-55, 56-65, >65 versus 26-35 years (reference category). Practice location size: 5000-20,000, 20,001-100,000, 100,001-500,000, >500,000 versus 5000 inhabitants (reference category). Professional experience: 6-10, 11-20, 21-30, >30 versus 1-5 years of experience (reference category). Practice type: Practice sharing, group practice, medical care center versus single practice (reference category). Current use of digital health solutions: Less than once per month, monthly, weekly, daily versus never (reference category).

In our final regression model, 4 variables were significantly associated with overall digital maturity (refer to Figure 5 for a visualization of the final regression model; a detailed overview of the coefficients for all models can be found in Multimedia Appendix 6). We found a significant association between overall digital maturity and the expected future use of digital health solutions (b=0.105, SE 0.051; β=.205; *P*=.04), the perceived digital affinity of medical assistants (b=0.1147, SE 0.044; β=.226; *P*=.001), respondents’ digital affinity (b=0.145, SE 0.041; β=.250; *P*=.001), and neuroticism (b=−0.216, SE 0.065; β=−0.238; *P*=.001). Overall, a higher expected likelihood of future digital health use, a higher digital affinity of medical assistants, a higher digital affinity of GPs, and lower neuroticism were associated with higher levels of overall digital maturity.

**Table 5:** Model parameters of the regression model predicting overall digital maturity (N=219).

Model number	Included variables^a^	*F* (*df*_M,_ *df*_R)_	*P* value	*R* ^2^	Δ*R*^2^	Δ*P* value
1	Demographics and practice-related characteristics	0.865 (16,202)	.61	0.064	0.064	.61
2	Demographics, practice-related characteristics, and digital health use	1.274 (21,197)	.20	0.120	0.056	.03
3	Demographics, practice-related characteristics, digital health use, and digital affinity	2.676 (23,195)	.001	0.240	0.120	.001
4	Demographics, practice-related characteristics, digital health use, digital affinity, and personality	3.029 (28,190)	.001	0.309	0.069	.003

^a^The models with their respective variables that were included in the different stages of our approach are as follows: model 1 (gender, age dummy coded, practice location size dummy coded, professional experience dummy coded, and practice type dummy coded), model 2 (model 1 variables, current use dummy coded, and expected future use); model 3 (model 2 variables, perceived digital affinity of medical assistants, and general practitioners’ digital affinity) and model 4 (model 3 variables, extraversion, agreeableness, conscientiousness, neuroticism, and openness).

## Discussion

### Principal Findings

The significance of digital maturity in ensuring a successful digital transformation and optimized service delivery has been proposed in numerous maturity models and corresponding studies[13,16,43,47,55]. However, limited research focuses on digital maturity in general practice settings [20,21]. Thus, this study sought to investigate digital maturity in general practices and specifically evaluate the relationship with GPs’ inherent characteristics. In our study, digital maturity was moderate and associated with several inherent characteristics. In our ANOVAs, we found differences in digital maturity based on gender, the expected future use of digital health solutions, the perceived digital affinity of medical assistants, GPs’ level of digital affinity, the level of extraversion, and the level of neuroticism. In addition, the expected future use of digital health solutions, the perceived digital affinity of medical assistants, GPs’ digital affinity, and neuroticism were significant predictors of digital maturity in the regression model.

### Comparison With Prior Work

#### Digital Maturity in General Practices

In line with previous findings investigating digital maturity in general practices across Europe [21], we found a moderate digital maturity of general practices (mean 3.31, SD 0.64) on our 5-point Likert-type scale that ranged from 1.60 to 4.94 in our sample. As pointed out by a study analyzing the readiness for digital health technologies in general practices in England [58], this variation highlights the need to investigate the various constituting dimensions of digital maturity and understand the factors influencing digital maturity in general practices.

Digital maturity is a multifaceted construct encompassing various technological and organizational dimensions beyond digital systems use [13]. Thus, there are oftentimes not only differences in the overall digital maturity between practices but also between the various underlying dimensions [21]. A recent study found the highest scores for overall digital systems use, followed by collective and individual resources and ability [21]. Likewise, our study’s subscales of *People, Skills, and Behavior* and *Governance and Management* received the highest scores. Both dimensions center around GPs’ capabilities and knowledge in using digital health solutions and leadership in digitalization. Interestingly, our study’s subscale *IT Capability*, which focuses on hardware and network resources and the actual use of digital health solutions, received a lower score, contrasting with previous findings [21]. A comparison between both studies should be interpreted cautiously, given the different underlying foci of digital maturity assessments. Although Teixeira et al [21] used a tool evaluating the digital maturity of electronic health record systems, our study assessed digital maturity more broadly. In addition, the difference in the findings might be attributable to geographical differences in health care delivery. In the United Kingdom, for example, digitalization in general practices is oftentimes managed and taken care of by dedicated practice managers [59]. In Germany, GPs mostly deal with digitalization-related topics themselves, which tends to be perceived as an add-on to the actual medical work [60,61], ultimately limiting adoption.

Given the underlying digital maturity indicators, the low *IT Capability* scores highlight a lack of digital health adoption in general practice. In line with this finding, the adoption of digital health solutions has been slow and cumbersome, as health care professionals perceive several technological, social, and organizational barriers to adoption [60,61], ultimately leading to resistance toward adoption in some cases [62]. However, as GPs’ adoption is one of the critical drivers for the success of digital health solutions [63], there is a potential for improvement regarding the use of digital health solutions.

The low scores in the *Patient-centered Care* dimension are especially surprising, as patient centeredness is proposed to be one of the leading quality indicators of digital health [64], benefiting patients’ disease knowledge, treatment adherence, self-management, and self-efficacy [65]. Consequently, digital health solutions were considered essential for empowering patients to participate actively in their health decisions [66]. Indeed, some studies found an association between patient-centered digital health solutions and patient empowerment and participation [67]. Accordingly, patient centeredness can somewhat be considered an outcome of successful digital health adoption. This aligns with our sample’s relatively low use of digital health solutions. As a result, patient centeredness might be a feature of digitally mature practices, but it is limited, given our sample’s moderate maturity. In contrast, our finding might be in line with the perceived additional workload required for digital health adoption and the resulting potential negative impact on the interaction between GPs and patients, which is oftentimes cited as a barrier to adoption [61].

#### Influencing Factors of Overall Digital Maturity in General Practices

Currently, only a few studies have investigated the factors that influence digital maturity in general practice settings [21]. Regarding gender and sex, evidence regarding digital maturity [21] and digital health use [24,68] are mixed. In line with our findings, some studies found that being male was associated with a higher digital maturity [21] and using digital health technology [24]. Studies also link higher electronic health record use to being female [68]. These mixed findings highlight that, in fact, there might be no difference in overall digital maturity based on gender or sex, that this difference is limited to some facets of digital maturity, or that covariates substantially influenced the effects found.

Although age is commonly identified as a factor promoting digital health adoption [24,69], our finding aligns with that of another study focusing on digital maturity in general practices [21]: in a multivariable linear regression model, age was not a significant predictor of digital maturity. The difference in findings compared with studies focusing on digital health adoption might be explained by the scope of research: digital maturity is a more faceted construct encompassing various dimensions [13]. Although adoption-related dimensions of digital maturity may be associated with age, other dimensions may not. This is in line with the study by Teixeira et al [21], who showed a higher odds ratio for respondents who were younger compared with respondents who were older only for some dimensions of digital maturity.

Besides the demographic variables mentioned, we did not find a significant difference in overall digital maturity based on various practice-related characteristics. Although the professional experience findings align with previous research [21] and mimic our results concerning age, the results regarding practice location are in contrast with previous literature. In their cross-country study, Teixeira et al [21] found that practicing in a rural setting was inversely associated with digital maturity (B=−0.25, 95% CI −0.43 to −0.08), but there was no substantial association with urban practice settings. One potential reason might be the underlying geographical scope of the studies and the corresponding broadband coverage. In 2021, the disparity in broadband uptake between urban and rural households was relatively small in Germany (1.8 percentage points difference) and below the sample average of Organisation for Economic Co-operation and Development (OECD) countries (3.83 percentage points) [70]. The slight difference in broadband uptake between urban and rural households in Germany and the higher OECD average might explain this contrast in findings. An Australian study further underlines this hypothesis, as Australia has an equally low disparity in broadband coverage below the OECD average [70]: they similarly found no substantial difference in telehealth use based on the rurality of the practice location [71].

Surprisingly, we did not find substantial differences in overall digital maturity based on the current use of digital health solutions. This contrasts with studies linking digital maturity to more frequent and long-term access to electronic health records [21] and with many digital maturity models proposing the implementation of digital health solutions as an essential dimension of digital maturity [13,14,16,45]. The findings might be attributed to our sample’s relatively low use of digital health solutions, as indicated by the relatively low scores for items relating to the actual use of individual digital health solutions in the *IT Capability* dimension of our digital maturity scale (eg, concerning e-prescription or digital appointment booking). As we found substantial differences in overall digital maturity based on the expected future use of digital health solutions, it might be beneficial to provide GPs with information highlighting the importance of digital health solutions, the latest advancements, and outlooks in digital health. This would act on the perceived lack of information and need for further information highlighted in studies on digital health adoption [60,72]. As our research design does not allow for causal inferences, another plausible explanation for this result may lie in the opposite direction: respondents with higher overall digital maturity are already more experienced with digital health solutions and might expect to continue using these in the future. However, the results of our linear regression analysis point toward the former explanation of the results, proposing the expected future use of digital health solutions as a significant predictor of overall digital maturity beyond demographics and practice-related characteristics.

GPs’ digital affinity in our study was comparable to a quota sample from the general public in larger German cities [33]. As our literature review pointed out, digital literacy is essential in maturity models [13,14,16]. In line with this, almost all interviewees in our expert interviews mentioned people- and skill-related indicators of technology use and perceived their relevance to be high. Taken together, our qualitative and quantitative results highlight the importance of digital skills for GPs and medical assistants. As digital affinity describes a person’s tendency to actively engage in intensive technology interaction and provides a first indication of the actual use of technical systems in everyday use settings [33], it is not surprising that we found a substantial difference in overall digital maturity based on both the perceived digital affinity of medical assistants and the respondents’ level of digital affinity. This association also holds in our regression model. In line with our findings, previous studies found a lack of digital skills to be a substantial barrier to digital health adoption [60,61,69]. Our finding further highlights the need for sufficient training of GPs and medical assistants regarding technology use, specifically digital health solutions [60,61], to enable the efficient use and management of these tools.

Although previous studies investigated the relationship between personality and digital health adoption [24], no study has examined the association between personality and digital maturity. This might be partially because of the inpatient focus of past research on digital maturity [13,20], where an individual physician has a rather subordinate role. Nevertheless, past research has already recognized that personality is an essential predictor of technology adoption and continued use of apps [25,26]. However, it has a relatively weak association with physicians’ digital health use [24]. Looking at the personality traits associated with the personality variables, our results on the association with overall digital maturity seem plausible. Extroverted individuals can be characterized as talkative, energetic, assertive, outgoing, and enthusiastic [73], and thus, they often take on leadership positions. This association is plausible, as digital maturity is, among others, characterized by transparent management and leadership [13]. Neuroticism represents tendencies such as being insecure, anxious, and hostile [73]. Respondents with higher neuroticism might also be more anxious and insecure about adopting digital health solutions, leading to a lower overall digital maturity. For conscientiousness, our findings might be owing to the overall high average in our sample. Given that we examined GPs, it is important to note that their profession inherently demands a significant amount of discipline, organization, exceptional diligence, and reliability. Thus, conscientiousness may not be associated with overall digital maturity, as this is a characteristic inherent to most physicians.

### Limitations

Although our study reveals important findings, it has some limitations. First, it needs to be noted that digital maturity encompasses various technological and organizational dimensions and focuses on the potential of general practices in the context of digitalization [13]. As there is a lack of evidence linking digital maturity to health care quality [1], a high level of digital maturity is not necessarily equivalent to a high quality of care. Thus, the findings of our study do not provide guidance on improving health care quality but can rather support GPs in their digitalization efforts by highlighting potential areas of improvement.

Second, as our research on the association of GPs’ inherent characteristics and digital maturity was exploratory, we did not use a psychometrically constructed and validated questionnaire to assess digital maturity. To our knowledge, no validated measure for digital maturity exists for general practice settings. Therefore, we based our questionnaire on a literature review of relevant digital maturity indicators in inpatient care settings and a validation of their relevance in expert interviews. This economical approach allows us to assess digital maturity based on existing models in inpatient care settings. As our findings are aligned with previous literature using different measures of digital maturity [21], we are confident that the measure used in our study is appropriate. Compared with the single technology-focused assessment in previous studies [21], a holistic maturity assessment covering all relevant digital maturity dimensions identified in the current literature [13] provides a more thorough picture. However, the measure used in our study should be validated in future studies. In addition, given the lack of a unified scale for digital maturity in general practices and thus different underlying measures, the comparison of our study with previous work is limited. Nevertheless, the consistency in findings might point to the reliability of the patterns identified and the validity of the scale used.

In addition, our literature review was limited to peer-reviewed articles published in German or English in the last 5 years and excluded gray literature. Thus, we might not have identified all the literature relevant to our research question and potentially encountered a publication bias. Nevertheless, as the COVID-19 pandemic has accelerated the adoption of digital health, we aimed at capturing more recent evolvements. To counter a potential publication bias and use an economic approach, we validated the findings of our literature review externally via expert interviews.

Although we found statistically significant differences in digital maturity based on various inherent characteristics, their practical relevance should be discussed. Although the absolute difference in digital maturity was small, the corresponding effect sizes were moderate to large. Thus, we are confident that besides the small absolute difference in digital maturity based on inherent characteristics, our findings still provide a basis to inform future research and practice, that is, the inclusion of GPs’ personal qualities when considering the digital maturity of their practice and designing interventions aimed at increasing digital maturity in general practice settings.

Owing to our cross-sectional study design, we cannot draw causal inferences regarding the relationship between GPs’ inherent characteristics and digital maturity. Although we found significant associations for some variables, we cannot assume a causal direction of the underlying effects. As personality can be considered a relatively stable characteristic, we can carefully interpret our findings so that the characteristics influence digital maturity. In addition, although inherent characteristics were associated with the current overall maturity level, it might be interesting to uncover the association with the advancement of digital maturity over time. Future research could take a longitudinal approach, following GPs along their journey and uncovering the associations between the advancement in digitalization and inherent characteristics. Specifically, it could be worthwhile to explore whether GPs’ inherent characteristics account for differences in the long-term digital maturity development of a practice. Although the proposed longitudinal approach is more effortful, its results provide insights into digital maturity development and could potentially provide a strong argument for tailoring approaches that support the digital agenda of GPs to their inherent characteristics.

Finally, our research focused on GPs in Germany, thus potentially limiting the generalizability of our findings to other countries. As GPs have a unique and central role in providing comprehensive and continuous health care services in European countries [22] and our findings are aligned with previous cross-country research [21], the findings from our study could be applied to health care systems with a similarly central role of GPs, for example, to the United Kingdom, the Netherlands, Scandinavian countries, Australia, New Zealand, or Canada. However, the findings might not apply to countries where GPs do not play a central role in health care provision. In addition, a potential application and replication in other health care systems must consider the different clinical practice features, health care delivery models, funding mechanisms, and specific organizational structures within the respective health care system as well as cultural differences that potentially shape GPs’ personalities differently.

Overall, we propose further research among a larger sample of GPs in Germany and other health care systems to validate the questionnaire used and extend the findings obtained. This could further enhance the value and practical relevance of our findings.

### Conclusions

As the research on digital maturity in general practices is still in its infancy, this study provides valuable insights into relevant influencing factors. It highlights that GPs have a pivotal role in the digital maturity of general practices and that there are significant differences in overall digital maturity based on GPs’ inherent characteristics. To our knowledge, this is the first study to specifically evaluate the association between personality characteristics and digital maturity. The results emphasize the relevance of personality characteristics for digital maturity in general practices by showing a substantial association between overall digital maturity, GPs’ digital affinity, and neuroticism. This association highlights the need for dedicated, targeted approaches based on inherent characteristics to support GPs in their digital agenda and ultimately drive their digital maturity. On the basis of our findings, regulators and other health care stakeholders could consider several measures to drive digital maturity and digital health adoption in general practices: (1) providing an outlook regarding ongoing trends and advancements in digital health, ultimately highlighting the potential and ubiquity of digital health in patient care in the future; (2) offering more dedicated training to GPs and medical assistants, ultimately enhancing their digital literacy; (3) prioritizing support for GPs who would particularly benefit from it owing to their inherent characteristics, for example, GPs who expect a low future use of digital health solutions and have a low digital affinity themselves and within their practice team; and (4) targeting approaches to meet the needs of different types of GPs’ rather than a one-size-fits-all approach based on GPs’ inherent characteristics associated with lower digital maturity, for example, by providing digital literacy training to GPs with low digital affinity or by providing campaigns that convey confidence and a feeling of trust in the digital transformation process to increase the digital maturity of GPs with high neuroticism.

## Appendix

Multimedia Appendix 1 Completed PRISMA-ScR (Preferred Reporting Items for Systematic Reviews and Meta-Analyses extension for Scoping Reviews) checklist.

Multimedia Appendix 2 Completed COREQ (Consolidated Criteria for Reporting Qualitative Research) checklist.

Multimedia Appendix 3 Translated semistructured interview guide.

Multimedia Appendix 4 Completed CHERRIES (Checklist for Reporting Results of Internet E-Surveys).

Multimedia Appendix 5 Translated survey questionnaire for general practitioners.

Multimedia Appendix 6 Overview of regression coefficients for the regression models predicting digital maturity.

## Abbreviations

CHERRIES: Checklist for Reporting Results of Internet E-Surveys
COREQ: Consolidated Criteria for Reporting Qualitative Research
GP: general practitioner
OECD: Organisation for Economic Co-operation and Development
PRISMA-ScR: Preferred Reporting Items for Systematic Reviews and Meta-Analyses extension for Scoping Reviews

## Data Availability

The data sets generated and analyzed during this study are available from the corresponding author on reasonable request.

## References

[1] Eden R, Burton-Jones A, Scott I, Staib A, Sullivan C (2018). Effects of eHealth on hospital practice: synthesis of the current literature. Aust Health Rev.

[2] Amarasingham R, Plantinga L, Diener-West M, Gaskin DJ, Powe NR (2009). Clinical information technologies and inpatient outcomes: a multiple hospital study. Arch Intern Med.

[3] Martin G, Clarke J, Liew F, Arora S, King D, Aylin P, Darzi A (2019). Evaluating the impact of organisational digital maturity on clinical outcomes in secondary care in England. NPJ Digit Med.

[4] Chaudhry B, Wang J, Wu S, Maglione M, Mojica W, Roth E, Morton SC, Shekelle PG (2006). Systematic review: impact of health information technology on quality, efficiency, and costs of medical care. Ann Intern Med.

[5] Lingg M, Lütschg V (2020). Health system stakeholders' perspective on the role of mobile health and its adoption in the Swiss health system: qualitative study. JMIR Mhealth Uhealth.

[6] Poissant L, Pereira J, Tamblyn R, Kawasumi Y (2005). The impact of electronic health records on time efficiency of physicians and nurses: a systematic review. J Am Med Inform Assoc.

[7] Li P, Lee GH, Kim SY, Kwon SY, Kim HR, Park S (2021). From diagnosis to treatment: recent advances in patient-friendly biosensors and implantable devices. ACS Nano.

[8] Wanderås MR, Abildsnes E, Thygesen E, Martinez SG (2023). Video consultation in general practice: a scoping review on use, experiences, and clinical decisions. BMC Health Serv Res.

[9] Ernsting C, Dombrowski SU, Oedekoven M, O Sullivan JL, Kanzler M, Kuhlmey A, Gellert P (2017). Using smartphones and health apps to change and manage health behaviors: a population-based survey. J Med Internet Res.

[10] Paré G, Raymond L, Castonguay A, Grenier Ouimet A, Trudel MC (2021). Assimilation of medical appointment scheduling systems and their impact on the accessibility of primary care: mixed methods study. JMIR Med Inform.

[11] Burmann A, Tischler M, Faßbach M, Schneitler S, Meister S (2021). The role of physicians in digitalizing health care provision: web-based survey study. JMIR Med Inform.

[12] Johnston DS (2017). Digital maturity: are we ready to use technology in the NHS?. Future Healthc J.

[13] Duncan R, Eden R, Woods L, Wong I, Sullivan C (2022). Synthesizing dimensions of digital maturity in hospitals: systematic review. J Med Internet Res.

[14] Vidal Carvalho J, Rocha Á, Abreu A (2019). Maturity of hospital information systems: most important influencing factors. Health Informatics J.

[15] Greenhalgh T, Rosen R, Shaw SE, Byng R, Faulkner S, Finlay T, Grundy E, Husain L, Hughes G, Leone C, Moore L, Papoutsi C, Pope C, Rybczynska-Bunt S, Rushforth A, Wherton J, Wieringa S, Wood GW (2021). Planning and evaluating remote consultation services: a new conceptual framework incorporating complexity and practical ethics. Front Digit Health.

[16] Kolukısa Tarhan A, Garousi V, Turetken O, Söylemez M, Garossi S (2020). Maturity assessment and maturity models in health care: a multivocal literature review. Digit Health.

[17] Gomes J, Romão M (2018). Information system maturity models in healthcare. J Med Syst.

[18] Mettler T, Pinto R (2018). Evolutionary paths and influencing factors towards digital maturity: an analysis of the status quo in Swiss hospitals. Technol Forecast Soc Change.

[19] van Poelgeest R, van Groningen JT, Daniels JH, Roes KC, Wiggers T, Wouters MW, Schrijvers G (2017). Level of digitization in Dutch hospitals and the lengths of stay of patients with colorectal cancer. J Med Syst.

[20] Neunaber T, Meister S (2023). Digital maturity and its measurement of general practitioners: a scoping review. Int J Environ Res Public Health.

[21] Teixeira F, Li E, Laranjo L, Collins C, Irving G, Fernandez MJ, Car J, Ungan M, Petek D, Hoffman R, Majeed A, Nessler K, Lingner H, Jimenez G, Darzi A, Jácome C, Neves AL (2022). Digital maturity and its determinants in general practice: a cross-sectional study in 20 countries. Front Public Health.

[22] Kringos DS, Boerma W, van der Zee J, Groenewegen P (2013). Europe's strong primary care systems are linked to better population health but also to higher health spending. Health Aff (Millwood).

[23] The European definition of general practice / family medicine. Wonca Europe.

[24] Zaresani A, Scott A (2020). Does digital health technology improve physicians' job satisfaction and work-life balance? A cross-sectional national survey and regression analysis using an instrumental variable. BMJ Open.

[25] Devaraj S, Easley RF, Crant JM (2008). Research note—how does personality matter? Relating the five-factor model to technology acceptance and use. Inf Syst Res.

[26] Su J, Dugas M, Guo X, Gao GG (2020). Influence of personality on mHealth use in patients with diabetes: prospective pilot study. JMIR Mhealth Uhealth.

[27] Tricco AC, Lillie E, Zarin W, O'Brien KK, Colquhoun H, Levac D, Moher D, Peters MD, Horsley T, Weeks L, Hempel S, Akl EA, Chang C, McGowan J, Stewart L, Hartling L, Aldcroft A, Wilson MG, Garritty C, Lewin S, Godfrey CM, Macdonald MT, Langlois EV, Soares-Weiser K, Moriarty J, Clifford T, Tunçalp Ö, Straus SE (2018). PRISMA extension for scoping reviews (PRISMA-ScR): checklist and explanation. Ann Intern Med.

[28] Tong A, Sainsbury P, Craig J (2007). Consolidated criteria for reporting qualitative research (COREQ): a 32-item checklist for interviews and focus groups. Int J Qual Health Care.

[29] Eysenbach G (2004). Improving the quality of web surveys: the Checklist for Reporting Results of Internet E-Surveys (CHERRIES). J Med Internet Res.

[30] Carvalho JV, Rocha Á, Abreu A (2016). Maturity models of healthcare information systems and technologies: a literature review. J Med Syst.

[31] VERBI software. MAXQDA.

[32] Kuckartz U, Rädiker S (2022). Qualitative Inhaltsanalyse. Methoden, Praxis, Computerunterstützung.

[33] Franke T, Attig C, Wessel D (2018). A personal resource for technology interaction: development and validation of the Affinity for Technology Interaction (ATI) scale. Int J Hum Comput Interact.

[34] Rammstedt B, John OP (2005). Kurzversion des Big Five Inventory (BFI-K): Entwicklung und Validierung eines ökonomischen Inventars zur Erfassung der fünf Faktoren der Persönlichkeit. Diagnostica.

[35] Leiner DJ (2019). Too fast, too straight, too weird: non-reactive indicators for meaningless data in internet surveys. Surv Res Method.

[36] Bais F, Schouten B, Toepoel V (2020). Investigating response patterns across surveys: do respondents show consistency in undesirable answer behaviour over multiple surveys?. Bull Sociol Methodol.

[37] IBM Corp IBM SPSS statistics for windows, version 29.0. IBM Corp.

[38] Tavakol M, Dennick R (2011). Making sense of Cronbach's alpha. Int J Med Educ.

[39] Field A (2017). Discovering Statistics Using IBM SPSS Statistics.

[40] Welch BL (1951). On the comparison of several mean values: an alternative approach. Biometrika.

[41] Agarwal A, Pritchard D, Gullett L, Amanti KG, Gustavsen G (2021). A quantitative framework for measuring personalized medicine integration into US healthcare delivery organizations. J Pers Med.

[42] Al-Kahtani N, Alruwaie S, Al-Zahrani BM, Abumadini RA, Aljaafary A, Hariri B, Alissa K, Alakrawi Z, Alumran A (2022). Digital health transformation in Saudi Arabia: a cross-sectional analysis using healthcare information and management systems society' digital health indicators. Digit Health.

[43] Brice S, Almond H (2020). Health professional digital capabilities frameworks: a scoping review. J Multidiscip Healthc.

[44] Carvalho JV, Rocha Á, van de Wetering R, Abreu A (2019). A maturity model for hospital information systems. J Bus Res.

[45] Chong J, Jason T, Jones M, Larsen D (2020). A model to measure self-assessed proficiency in electronic medical records: validation using maturity survey data from Canadian community-based physicians. Int J Med Inform.

[46] Grooten L, Borgermans L, Vrijhoef HJ (2018). An instrument to measure maturity of integrated care: a first validation study. Int J Integr Care.

[47] Khanbhai M, Flott K, Manton D, Harrison-White S, Klaber R, Darzi A, Mayer E (2021). Identifying factors that promote and limit the effective use of real-time patient experience feedback: a mixed-methods study in secondary care. BMJ Open.

[48] Kose I, Rayner J, Birinci S, Ulgu MM, Yilmaz I, Guner S (2020). Adoption rates of electronic health records in Turkish Hospitals and the relation with hospital sizes. BMC Health Serv Res.

[49] Kouroubali A, Papastilianou A, Katehakis DG (2019). Preliminary assessment of the interoperability maturity of healthcare digital services vs public services of other sectors. Stud Health Technol Inform.

[50] Krasuska M, Williams R, Sheikh A, Franklin BD, Heeney C, Lane W, Mozaffar H, Mason K, Eason S, Hinder S, Dunscombe R, Potts HW, Cresswell K (2020). Technological capabilities to assess digital excellence in hospitals in high performing health care systems: international eDelphi exercise. J Med Internet Res.

[51] Martin G, Arora S, Shah N, King D, Darzi A (2020). A regulatory perspective on the influence of health information technology on organisational quality and safety in England. Health Informatics J.

[52] Orenstein EW, Muthu N, Weitkamp AO, Ferro DF, Zeidlhack MD, Slagle J, Shelov E, Tobias MC (2019). Towards a maturity model for clinical decision support operations. Appl Clin Inform.

[53] Pumplun L, Fecho M, Wahl N, Peters F, Buxmann P (2021). Adoption of machine learning systems for medical diagnostics in clinics: qualitative interview study. J Med Internet Res.

[54] Wherton J, Greenhalgh T, Shaw SE (2021). Expanding video consultation services at pace and scale in Scotland during the COVID-19 pandemic: national mixed methods case study. J Med Internet Res.

[55] Williams PA, Lovelock B, Cabarrus T, Harvey M (2019). Improving digital hospital transformation: development of an outcomes-based infrastructure maturity assessment framework. JMIR Med Inform.

[56] Woods L, Eden R, Pearce A, Wong YC, Jayan L, Green D, McNeil K, Sullivan C (2022). Evaluating digital health capability at scale using the digital health indicator. Appl Clin Inform.

[57] Gesundheitsdaten. Kassenärztliche Bundesvereinigung.

[58] Hammerton M, Benson T, Sibley A (2022). Readiness for five digital technologies in general practice: perceptions of staff in one part of southern England. BMJ Open Qual.

[59] Hanna L, May C, Fairhurst K (2011). Non-face-to-face consultations and communications in primary care: the role and perspective of general practice managers in Scotland. Inform Prim Care.

[60] Jacob C, Sanchez-Vazquez A, Ivory C (2020). Social, organizational, and technological factors impacting clinicians' adoption of mobile health tools: systematic literature review. JMIR Mhealth Uhealth.

[61] Gagnon MP, Ngangue P, Payne-Gagnon J, Desmartis M (2016). m-Health adoption by healthcare professionals: a systematic review. J Am Med Inform Assoc.

[62] Choi WS, Park J, Choi JY, Yang JS (2019). Stakeholders' resistance to telemedicine with focus on physicians: utilizing the Delphi technique. J Telemed Telecare.

[63] Gagnon MP, Desmartis M, Labrecque M, Car J, Pagliari C, Pluye P, Frémont P, Gagnon J, Tremblay N, Légaré F (2012). Systematic review of factors influencing the adoption of information and communication technologies by healthcare professionals. J Med Syst.

[64] Ibrahim MS, Mohamed Yusoff H, Abu Bakar YI, Thwe Aung MM, Abas MI, Ramli RA (2022). Digital health for quality healthcare: a systematic mapping of review studies. Digit Health.

[65] Brands MR, Gouw SC, Beestrum M, Cronin RM, Fijnvandraat K, Badawy SM (2022). Patient-centered digital health records and their effects on health outcomes: systematic review. J Med Internet Res.

[66] Hägglund M, Cajander Å, Rexhepi H, Kane B (2022). Editorial: personalized digital health and patient-centric services. Front Comput Sci.

[67] Leonardsen AC, Hardeland C, Helgesen AK, Grøndahl VA (2020). Patient experiences with technology enabled care across healthcare settings- a systematic review. BMC Health Serv Res.

[68] Holanda AA, do Carmo E Sá HL, Vieira AP, Catrib AM (2012). Use and satisfaction with electronic health record by primary care physicians in a health district in Brazil. J Med Syst.

[69] O'Donnell A, Kaner E, Shaw C, Haighton C (2018). Primary care physicians' attitudes to the adoption of electronic medical records: a systematic review and evidence synthesis using the clinical adoption framework. BMC Med Inform Decis Mak.

[70] Disparity in broadband uptake between urban and rural households. Organisation for Economic Co-operation and Development Going Digital Toolkit.

[71] Scott A, Bai T, Zhang Y (2021). Association between telehealth use and general practitioner characteristics during COVID-19: findings from a nationally representative survey of Australian doctors. BMJ Open.

[72] Dahlhausen F, Zinner M, Bieske L, Ehlers JP, Boehme P, Fehring L (2021). Physicians' attitudes toward prescribable mHealth apps and implications for adoption in Germany: mixed methods study. JMIR Mhealth Uhealth.

[73] McCrae RR, Costa PT Jr (1987). Validation of the five-factor model of personality across instruments and observers. J Pers Soc Psychol.

