# Supplementary material for: Big 5 Personality Traits and Individual- and Practice-Related Characteristics as Influencing Factors of Digital Maturity in General Practices: Quantitative Web-Based Survey Study
Source: J Med Internet Res. 2024 Jan 22;26:e52085. doi: 10.2196/52085 (PMC10845021; doi:10.2196/52085)
Supplement: Multimedia Appendix 1 [file jmir_v26i1e52085_app1.docx]

**Multimedia Appendix 1: Completed PRISMA-ScR (Preferred Reporting Items for Systematic Reviews and Meta-Analyses extension for Scoping Reviews) checklist.**

| **SECTION** | **ITEM** | **PRISMA-ScR CHECKLIST ITEM** | **REPORTED IN SECTION** |
| --- | --- | --- | --- |
| **TITLE** | | | |
| Title | 1 | Identify the report as a scoping review. | n/a |
| **ABSTRACT** | | | |
| Structured summary | 2 | Provide a structured summary that includes (as applicable): background, objectives, eligibility criteria, sources of evidence, charting methods, results, and conclusions that relate to the review questions and objectives. | Background: Vast research has already shown the potential value of digital health and the importance of general practitioners' adoption regarding the success of these technologies. In this context, several maturity models have been developed for inpatient care, especially in hospitals. However, little is known about the dimensions relevant to digital maturity in general practices. Thus, we aimed to identify and synthesize relevant dimensions of digital maturity that can be adapted to general practices.  Methods: For our review, we searched the PubMed and PsycINFO databases on 20 February 2023 and manually de-duplicated the results. We narrowed initially identified citations to studies published in English or German between January 2018 and December 2022 to account for more recent research findings. We included all articles that focused on middle to high-income countries, digital health, and digital maturity in individual care organizations providing outpatient care that described the development, application, validation, or synthesis of indicators, frameworks, or assessments of digital maturity. We included only peer-reviewed articles, as we aimed to identify validated indicators of digital maturity. Evidence from the included studies was synthesized by extracting and grouping potentially relevant indicators of digital maturity.  Findings: Of 554 records initially identified in the two databases, 21 were eligible. Most studies were quantitative (8/21; 38%) and focused on maturity assessment (9/21; 43%) in hospitals (9/21; 43%). Seventeen distinct maturity models were presented and utilized in the studies, with only around half looking at people- and skill-related indicators of digital maturity (10/17; 58%). Of these, technology usage (8/10; 80%) was the most cited determinant of digital maturity. All other determinants in this category were only present in 3 of the 17 maturity models (18%), covering the aspects of education and training, knowledge management, and individual competence.  Interpretation: Many studies investigated the digital maturity of healthcare systems. However, the current maturity models mainly cover technological and management-related digital maturity determinants and focus on the inpatient care context. To date, no maturity model investigated characteristics of physicians’ as relevant aspects of digital maturity. |
| **INTRODUCTION** | | | |
| Rationale | 3 | Describe the rationale for the review in the context of what is already known. Explain why the review questions/objectives lend themselves to a scoping review approach. | The rapid global uptake of digital health technologies requires healthcare professionals to decide on their digital health agenda. Vast research has already shown the potential value of digital health and the importance of clinicians’ adoption regarding the success of these technologies. Since general practitioners are patients' first point of contact, identifying factors relevant to their digital maturity is crucial. Many theoretical maturity models have been developed and applied to different healthcare contexts. However, most of these models have been applied in the inpatient care context. Of the maturity models postulated, only a few look into people-related indicators of digital maturity. Identifying the dimensions and indicators relevant to digital maturity in general practice settings will drive maturity assessments in primary care. |
| Objectives | 4 | Provide an explicit statement of the questions and objectives being addressed with reference to their key elements (e.g., population or participants, concepts, and context) or other relevant key elements used to conceptualize the review questions and/or objectives. | Our review aimed at identifying currently postulated dimensions and indicators of digital maturity in individual healthcare organizations more broadly. With this, a subsequent study would allow us to (1) create transparency on the current digital maturity of primary care physicians in Germany and (2) assess the role of personality traits as influencing factors of digital maturity. |
| **METHODS** | | | |
| Protocol and registration | 5 | Indicate whether a review protocol exists; state if and where it can be accessed (e.g., a Web address); and if available, provide registration information, including the registration number. | As this review was part of a more comprehensive study approach, we did not prepare, register, and publish a review protocol. |
| Eligibility criteria | 6 | Specify characteristics of the sources of evidence used as eligibility criteria (e.g., years considered, language, and publication status), and provide a rationale. | see ‘Literature Review’ |
| Information sources | 7 | Describe all information sources in the search (e.g., databases with dates of coverage and contact with authors to identify additional sources), as well as the date the most recent search was executed. | see ‘Literature Review’ |
| Search | 8 | Present the full electronic search strategy for at least 1 database, including any limits used, such that it could be repeated. | To identify potentially relevant articles for our research, we used the following Boolean search string in both databases: ("maturity model") OR ("maturity assessment") OR ("digital capabilit*") OR ("digital maturity") OR ("digital excellence"). We applied the search string to the Title/Abstract fields in PubMed and the full-text fields in PsycINFO. We developed the search terms following other reviews on digital maturity in the digital health context and discussed and aligned them between the authors. We narrowed initially identified citations to studies published in English or German between January 2018 and December 2022 to account for more recent research findings. |
| Selection of sources of evidence | 9 | State the process for selecting sources of evidence (i.e., screening and eligibility) included in the scoping review. | see ‘Literature Review’ |
| Data charting process | 10 | Describe the methods of charting data from the included sources of evidence (e.g., calibrated forms or forms that have been tested by the team before their use, and whether data charting was done independently or in duplicate) and any processes for obtaining and confirming data from investigators. | We developed a data extraction sheet listing all the relevant data we needed to extract from the eligible studies. According to the sheet, LW extracted all data from eligible studies. All data were extracted as reported in the studies and compared after extraction to harmonize wording across studies. |
| Data items | 11 | List and define all variables for which data were sought and any assumptions and simplifications made. | We sought data for all indicators and dimensions of digital maturity utilized in the eligible studies, i.e., that were reported as part of the maturity model presented or as scores in a maturity assessment. In addition, we sought data on study-related characteristics. We extracted information from each eligible report on (1) the report itself (including author, publication year, journal, and publication identifier); (2) the study itself (including sample characteristics, sample size, study location, and healthcare setting); (3) the research design (including the type of research, i.e., framework development vs. maturity assessment, and study design); (4) the intervention (i.e., the type of digital health solution under consideration) and (5) the underlying digital health maturity model presented or utilized in the study (including the model name, number of stages, model description, and indicators). |
| Critical appraisal of individual sources of evidence | 12 | If done, provide a rationale for conducting a critical appraisal of included sources of evidence; describe the methods used and how this information was used in any data synthesis (if appropriate). | We did not conduct a dedicated critical appraisal of included studies as we aimed to identify indicators of digital maturity as presented in multiple maturity models and only included evidence from more than one model and aimed to validate the results of our literature review in qualitative expert interviews with general practitioners, ensuring relevance and completeness of our results. |
| Synthesis of results | 13 | Describe the methods of handling and summarizing the data that were charted. | see ‘Literature Review’ |
| **RESULTS** | | | |
| Selection of sources of evidence | 14 | Give numbers of sources of evidence screened, assessed for eligibility, and included in the review, with reasons for exclusions at each stage, ideally using a flow diagram. | see ‘Characterizing Digital Maturity in General Practices (Literature Review and Expert Interview Results)’ and ‘Figure 2’ |
| Characteristics of sources of evidence | 15 | For each source of evidence, present characteristics for which data were charted and provide the citations. | see ‘Characterizing Digital Maturity in General Practices (Literature Review and Expert Interview Results)’  The detailed characteristics of included studies and the extracted data mentioned above can be obtained from the corresponding author upon request and is not presented in detail. |
| Critical appraisal within sources of evidence | 16 | If done, present data on critical appraisal of included sources of evidence (see item 12). | n/a |
| Results of individual sources of evidence | 17 | For each included source of evidence, present the relevant data that were charted that relate to the review questions and objectives. | See Characterizing Digital Maturity in General Practices (Literature Review and Expert Interview Results)’ |
| Synthesis of results | 18 | Summarize and/or present the charting results as they relate to the review questions and objectives. | See Characterizing Digital Maturity in General Practices (Literature Review and Expert Interview Results)’ and ‘Figure 3’ |
| **DISCUSSION** | | | |
| Summary of evidence | 19 | Summarize the main results (including an overview of concepts, themes, and types of evidence available), link to the review questions and objectives, and consider the relevance to key groups. | see ‘Characterizing Digital Maturity in General Practices (Literature Review and Expert Interview Results)’ |
| Limitations | 20 | Discuss the limitations of the scoping review process. | First, as we limited our search to peer-reviewed articles published in German or English and excluded grey literature, we might not have identified all literature relevant to our research question and potentially encountered a publication bias. In addition, our inclusion and exclusion criteria defined before our search also limited our approach.  Second, as we further limited our search to articles published in the last five years, we might have missed older maturity models. Thus, we might not have captured all determinants proposed in the literature. As the COVID-19 pandemic has accelerated the adoption of digital health, we aimed at capturing more recent evolvements.  Third, as our review was part of a more extensive study, our search, data extraction, and synthesis were solely conducted by one author and not validated by a second reviewer. We are confident, that this would not have changed our synthesis of results as we validated these externally via expert interviews. |
| Conclusions | 21 | Provide a general interpretation of the results with respect to the review questions and objectives, as well as potential implications and/or next steps. | Findings from our review suggest that there is already a substantial body of research on digital maturity in inpatient care. However, some critical aspects are worth further exploration. As no study today has explored the personality of general practitioners as a potentially relevant influencing factor of the digital maturity of the practice, we aim to shed light on this association in our subsequent study design. We believe that exploring personality as a potential influencing factor of digital maturity is particularly important in general practices as (1) some maturity models already highlight the importance of individual capabilities, specifically concerning technology usage, and (2) digitalization in general practices is mainly driven by the general practitioner himself. |
| **FUNDING** | | | |
| Funding | 22 | Describe sources of funding for the included sources of evidence, as well as sources of funding for the scoping review. Describe the role of the funders of the scoping review. | As this research is part of a doctoral thesis at Witten/Herdecke University, it has not received any funding. |
