# Supplementary material for: Big 5 Personality Traits and Individual- and Practice-Related Characteristics as Influencing Factors of Digital Maturity in General Practices: Quantitative Web-Based Survey Study
Source: J Med Internet Res. 2024 Jan 22;26:e52085. doi: 10.2196/52085 (PMC10845021; doi:10.2196/52085)
Supplement: Multimedia Appendix 2 [file jmir_v26i1e52085_app2.docx]

**Multimedia Appendix 2: Completed Consolidated Criteria for Reporting Qualitative Research (COREQ) checklist.**

| **DOMAIN** | **ITEM** | **GUIDE QUESTIONS / DESCRIPTIONS** | **REPORTED IN SECTION** |
| --- | --- | --- | --- |
| **DOMAIN 1: RESEARCH TEAM AND REFLEXIVITY** | | | |
| ***Personal characteristics*** | | | |
| Interviewer/facilitator | 1 | Which author/s conducted the interview or focus group? | see ‘Authors’ Contributions’ |
| Credentials | 2 | What were the researcher's credentials? (e.g., PhD, MD) | LW holds a Master of Science in Psychology. |
| Occupation | 3 | What was their occupation at the time of the study? | LW is a Ph.D. candidate. |
| Gender | 4 | Was the researcher male or female? | LW is female. |
| Experience and training | 5 | What experience or training did the researcher have? | LW was experienced in conducting interviews based on her educational background in psychology and her professional background in strategy consulting. |
| ***Relationship with participants*** | | | |
| Relationship established | 6 | Was a relationship established prior to study commencement? | There was no relationship established to the participants prior to the study. |
| Participant knowledge of the interviewer | 7 | What did the participants know about the researcher? (e.g., personal goals, reasons for doing the research) | In the beginning of the interview, participants were informed about the educational and professional background of the researcher, and the overall objectives and aims of the research. |
| Interviewer characteristics | 8 | What characteristics were reported about the interviewer/facilitator? (e.g., bias, assumptions, reasons, and interests in the research topic) | Participants were informed that the interest in the research topic was based on the researcher’s professional background. |
| **DOMAIN 2: STUDY DESIGN** | | | |
| ***Theoretical framework*** | | | |
| Methodological orientation and theory | 9 | What methodological orientation was stated to underpin the study? (e.g., grounded theory, discourse analysis, ethnography, phenomenology, content analysis) | see ‘Study Design’ and ‘Expert Interviews’ |
| ***Participant selection*** | | | |
| Sampling | 10 | How were participants selected? (e.g., purposive, convenience, consecutive, snowball) | see ‘Expert Interviews’ |
| Method of approach | 11 | How were participants approached? (e.g., face-to-face, telephone, mail, email) | see ‘Expert Interviews’ |
| Sample size | 12 | How many participants were in the study? | see ‘Expert Interviews’ |
| Non-participation | 13 | How many people refused to participate or dropped out? Reasons? | Initially we approached 26 general practitioners, of which 15 did not respond to our interview enquiry. One additional general practitioner declined to be interviewed due to a lack of time and high workload. |
| ***Setting*** | | | |
| Setting of data collection | 14 | Where was the data collected? (e.g., home, clinic, workplace) | see ‘Expert Interviews’ |
| Presence of non-participants | 15 | Was anyone else present besides the participants and researchers? | see ‘Expert Interviews’ |
| Description of sample | 16 | What are important characteristics of the sample? (e.g., demographic data, date) | see ‘Expert Interviews’  As we purposively sampled participants, we were able to include diverse age ranges and work experience levels as well as locations and practice types. However only 20% of interviewees were female. On average, participants were 53 years old and have worked as a general practitioner for 18 years in a city with 105k inhabitants. Four general practitioners worked in a single practice, five worked in a group practice and one worked in a medical care center. |
| ***Data collection*** | | | |
| Interview guide | 17 | Were questions, prompts, guides provided by the authors? Was it pilot tested? | see ‘Expert Interviews’  We utilized a semi-structured interview guide for the interviews to allow for flexibility yet achieve standardization of the interview procedure. |
| Repeat interviews | 18 | Were repeat interviews carried out? If yes, how many? | No repeat interviews were carried out. |
| Audio/visual recording | 19 | Did the research use audio or visual recording to collect the data? | see ‘Expert Interviews’ |
| Field notes | 20 | Were field notes made during and/or after the interview or focus group? | No field notes were made during the interviews. However, a short summary of the interview was written after transcription. |
| Duration | 21 | What was the duration of the interviews or focus group? | see ‘Expert Interviews’ |
| Data saturation | 22 | Was data saturation discussed? | see ‘Expert Interviews’  We planned to interview between 8 and 15 general practitioners based on data saturation. This was achieved, i.e., no new content on top of the derived themes based on the literature research emerged by the time we interviewed 10 general practitioners. |
| Transcripts returned | 23 | Were transcripts returned to participants for comment and/or correction? | We did not return transcripts to participants. |
| **DOMAIN 3: ANALYSIS AND FINDINGS** | | | |
| ***Data analysis*** | | | |
| Number of data coders | 24 | How many data coders coded the data? | see ‘Authors’ Contributions’ |
| Description of the coding tree | 25 | Did authors provide a description of the coding tree? | see ‘Expert Interviews’ |
| Derivation of themes | 26 | Were themes identified in advance or derived from the data? | see ‘Expert Interviews’ |
| Software | 27 | What software, if applicable, was used to manage the data? | see ‘Expert Interviews’ |
| Participant checking | 28 | Did participants provide feedback on the findings? | We did not ask for feedback on the findings from participants. |
| ***Reporting*** | | | |
| Quotations presented | 29 | Were participant quotations presented to illustrate the themes/findings? Was each quotation identified? (e.g., participant number) | We do not present participant quotes. |
| Data and findings consistent | 30 | Was there consistency between the data presented and the findings? | Data presented and findings are consistent. |
| Clarity of major themes | 31 | Were major themes clearly presented in the findings? | see ‘Characterizing Digital Maturity in General Practices (Literature Review and Expert Interview Results)’  The section provides a quantitative overview of interview themes and their contrast with literature review results. We do not discriminate between major or minor themes. |
| Clarity of minor themes | 32 | Is there a description of diverse cases or discussion of minor themes? | see ‘Characterizing Digital Maturity in General Practices (Literature Review and Expert Interview Results)’ |
