# Supplementary material for: Big 5 Personality Traits and Individual- and Practice-Related Characteristics as Influencing Factors of Digital Maturity in General Practices: Quantitative Web-Based Survey Study
Source: J Med Internet Res. 2024 Jan 22;26:e52085. doi: 10.2196/52085 (PMC10845021; doi:10.2196/52085)
Supplement: Multimedia Appendix 3 [file jmir_v26i1e52085_app3.docx]

**Multimedia Appendix 3: Translated semistructured interview guide.**

*Introduction of the interviewer and the research question*

*Introduction to the interview format and topics to be covered*

*Brief definition of the terms ‘digital maturity’ and ‘digital health solutions’*

***Part 1: Experience with digital health solutions* ___________________________**

- Could you tell me a little about yourself (your age, professional experience, place of work)?
- Could you tell me a little about your practice, including the type of practice and the patient population (statutory vs. privately health-insured patients)?
- Do you have any experience with digital health solutions (e.g., video consultations, electronic patient records, e-prescribing)?
- If so, which digital health solutions have you implemented in your practice?

***Part 2: Perspectives on indicators of digital maturity* ______________________**

- How would you rate the digital maturity of your practice on a scale from 1 (low maturity) to 5 (high maturity)?
- Which criteria or factors did you apply in arriving at this assessment?
- Which additional influencing factors do you consider relevant concerning the digital maturity of general practices?
- On a scale of 1 (not relevant at all) to 10 (very relevant), how would you rate the relevance of indicators in the following dimensions for the digital maturity of general practices?
  1. *Governance & Management*, i.e., the extent to which practices have mechanisms and structures in place to ensure data security, quality of care, sufficient resources, and efficiency.
  2. *IT Capability*, i.e., the extent to which practices are equipped with appropriate and effective IT infrastructure and have implemented digital systems and services.
  3. *People, Skills & Behavior*, i.e., the extent to which practices and practice staff are digitally literate and motivated to use digital systems.
  4. *Interoperability*, i.e., the extent to which data and information can be shared between internal systems and external partners.
  5. *Strategy*, i.e., the extent to which practices pursue strategic goals, including in the digital domain.
  6. *Data Analytics*, i.e., the extent to which practices use data for effective decision-making.
  7. *Patient-centered Care*, i.e., the extent to which practices digitally share information with patients and adapt digital systems to them.
- In the aforementioned dimensions or beyond – which other indicators do you think are relevant when assessing the digital maturity of general practices?

*Part 3: Perceived barriers to the adoption of digital health solutions*

*Part 4: Preferable strategies to improve digital health adoption*

*Conclusion and goodbye*
