# Supplementary material for: Big 5 Personality Traits and Individual- and Practice-Related Characteristics as Influencing Factors of Digital Maturity in General Practices: Quantitative Web-Based Survey Study
Source: J Med Internet Res. 2024 Jan 22;26:e52085. doi: 10.2196/52085 (PMC10845021; doi:10.2196/52085)
Supplement: Multimedia Appendix 4 [file jmir_v26i1e52085_app4.docx]

**Multimedia Appendix 4: Completed Checklist for Reporting Results of Internet E-Surveys (CHERRIES).**

| **ITEM CATEGORY** | **ITEM** | **EXPLANATION** | **REPORTED IN SECTION** |
| --- | --- | --- | --- |
| **DESIGN** | | | |
| Describe survey design | 1 | Describe target population, sample frame. Is the sample a convenience sample? (In “open” surveys this is most likely.) | see ‘Survey Design’ |
| **IRB (Institutional Review Board) APPROVAL AND INFORMED CONSENT PROCESS** | | | |
| IRB approval | 2 | Mention whether the study has been approved by an IRB. | see ‘Study Design’ |
| Informed consent | 3 | Describe the informed consent process. Where were the participants told the length of time of the survey, which data were stored and where and for how long, who the investigator was, and the purpose of the study? | see ‘Survey Design’ |
| Data protection | 4 | If any personal information was collected or stored, describe what mechanisms were used to protect unauthorized access. | Data were anonymized following the principle of k-anonymity and stored solely on the Witten/Herdecke University server. We did not collect any personal information that could be tied back to individual respondents. |
| **DEVELOPMENT AND PRE-TESTING** | | | |
| Development and testing | 5 | State how the survey was developed, including whether the usability and technical functionality of the electronic questionnaire had been tested before fielding the questionnaire. | see ‘Study Design’ and ‘Pretest and Recruitment’ |
| **RECRUITMENT PROCESS AND DESCRIPTION OF THE SAMPLE HAVING ACCESS TO THE QUESTIONNAIRE** | | | |
| Open survey versus closed survey | 6 | An “open survey” is a survey open for each visitor of a site, while a closed survey is only open to a sample which the investigator knows (password-protected survey). | see ‘Pretest and Recruitment’ |
| Contact mode | 7 | Indicate whether or not the initial contact with the potential participants was made on the Internet. (Investigators may also send out questionnaires by mail and allow for Web-based data entry.) | see ‘Pretest and Recruitment’ |
| Advertising the survey | 8 | How/where was the survey announced or advertised? Some examples are offline media (newspapers), or online (mailing lists – If yes, which ones?) or banner ads (Where were these banner ads posted and what did they look like?). It is important to know the wording of the announcement as it will heavily influence who chooses to participate. Ideally the survey announcement should be published as an appendix. | see ‘Pretest and Recruitment’  The announcements used for recruitment varied slightly in length and style to match the audience and channel. Overall, we provided the different channels with a formal digital letter signed by the research team as an official invitation to participate in the survey, a digital flyer containing the most relevant information about the survey (i.e., research question, target group, survey duration), and the respective survey link. The formal letter included the following message (translated English version): "Dear Sir or Madam, The Chair of Health Informatics at Witten/Herdecke University is currently looking for participants for a web-based survey entitled 'digitalization in general practice settings - influencing factors and perceived barriers'. In the research project, the team investigates the degree of digitalization in German general practices, perceived barriers, and potential strategies for improvement concerning the adoption of digital health solutions. The survey, which takes about 15 minutes to complete, is aimed at practicing general practitioners with a practice location in Germany, regardless of their experience with digital health solutions. You are welcome to follow this link to participate in the survey: [survey link]. If you have any questions or suggestions, please feel free to contact the chair team [mail address].".  On LinkedIn, the survey was shared with the following message (translated English version): "Digitalization in general practices and the human factor - obstacle or success factor? The Chair of Health Informatics at Witten/Herdecke University currently investigates this question in a short web-based survey. Our research aims to assess the degree of digitalization of German general practices, record perceived barriers to adopting digital health solutions, and further investigate the relationship with personal characteristics. The 15-minute survey targets practicing general practitioners with practices in Germany, regardless of their experience with digital health solutions. You are welcome to participate at: [survey link].". |
| **SURVEY ADMINISTRATION** | | | |
| Web/E-Mail | 9 | State the type of e-survey (e.g., one posted on a Web site, or one sent out through e-mail). If it is an e-mail survey, were the responses entered manually into a database, or was there an automatic method for capturing responses? | The survey was administered solely through the web. We utilized the web-based survey tool LimeSurvey to create and launch the survey. |
| Context | 10 | Describe the Web site (for mailing list/newsgroup) in which the survey was posted. What is the Web site about, who is visiting it, what are visitors normally looking for? Discuss to what degree the content of the Web site could pre-select the sample or influence the results. For example, a survey about vaccination on an anti-immunization Web site will have different results from a Web survey conducted on a government Web site | n/a |
| Mandatory/voluntary | 11 | Was it a mandatory survey to be filled in by every visitor who wanted to enter the Web site, or was it a voluntary survey? | see ‘Pretest and Recruitment’ |
| Incentives | 12 | Were any incentives offered (e.g., monetary, prizes, or non-monetary incentives such as an offer to provide the survey results)? | see ‘Pretest and Recruitment’ |
| Time/date | 13 | In what timeframe were the data collected? | see ‘Pretest and Recruitment’ |
| Randomization of items or questionnaires | 14 | To prevent biases items can be randomized or alternated. | We did not randomize items as we chose the sequence of questions based on general questionnaire design guidelines. We asked participants to answer questions on their demographics, practice-related characteristics, digital health usage-related variables, digital affinity-related variables, and personality-related variables first (i.e., independent variables), before they had to answer the remaining items for dependent variables (i.e., digital maturity, adoption barriers, measures to support adoption). |
| Adaptive questioning | 15 | Use adaptive questioning (certain items, or only conditionally displayed based on responses to other items) to reduce number and complexity of the questions. | As we partially utilized existing instruments, we did not use adaptive questioning in our survey. |
| Number of items | 16 | What was the number of questionnaire items per page? The number of items is an important factor for the completion rate. | see ‘Measures’  The total number of questions was 17, with demographics, practice-related characteristics, and digital health usage-related variables containing nine single-choice questions, digital affinity and personality each containing one Likert-type scale, and the remaining three sections each being spread across two survey pages and thus containing two Likert-type scales each. Across all questions our survey consisted of 116 items. |
| Number of screens (pages) | 17 | Over how many pages was the questionnaire distributed? The number of items is an important factor for the completion rate. | In general, sections (1) to (3) were displayed on separate questionnaire pages, while sections (4) to (6) were each split into two pages to reduce the number of items per page and allow for a more user-friendly completion of the questionnaire. In total, the survey was distributed over 12 pages, with nine questionnaire pages covering the different sections, one welcome and goodbye screen, and one screen to provide informed consent. |
| Completeness check | 18 | It is technically possible to do consistency or completeness checks before the questionnaire is submitted. Was this done, and if “yes”, how (usually JAVAScript)? An alternative is to check for completeness after the questionnaire has been submitted (and highlight mandatory items). If this has been done, it should be reported. All items should provide a non-response option such as “not applicable” or “rather not say”, and selection of one response option should be enforced. | We utilized forced responses for the first three sections and soft-forced-response (i.e., reminders to provide complete answers) for the latter. To enforce the selection of one response option and not undermine the forced response of the question, the survey did not provide an "I don't know" option. We applied both strategies to gain complete, meaningful responses, even though this might have led to a lower response rate. |
| Review step | 19 | State whether respondents were able to review and change their answers (e.g., through a Back button or a Review step which displays a summary of the responses and asks the respondents if they are correct). | We decided not to include a "back" button for respondents to review and change their responses to counter a potential social desirability effect that might cause them to change their responses after completing subsequent questions. |
| **RESPONSE RATES** | | | |
| Unique site visitors | 20 | If you provide view rates or participation rates, you need to define how you determined a unique visitor. There are different techniques available, based on IP addresses or cookies or both. | see ‘Data Cleaning and Analysis’ and ‘Figure 1’ |
| View rate (Ratio of unique survey visitors/unique site visitors) | 21 | Requires counting unique visitors to the first page of the survey, divided by the number of unique site visitors (not page views!). It is not unusual to have view rates of less than 0.1 % if the survey is voluntary. | n/a |
| Participation rate (ratio of those who agreed to participate/unique first survey page visitors) | 22 | Count the unique number of people who filled in the first survey page (or agreed to participate, for example by checking a checkbox), divided by visitors who visit the first page of the survey (or the informed consents page, if present). This can also be called “recruitment” rate. | see ‘Data Cleaning and Analysis’ and ‘Figure 1’ |
| Completion rate (ratio of users who finished the survey/users who agreed to participate) | 23 | The number of people submitting the last questionnaire page, divided by the number of people who agreed to participate (or submitted the first survey page). This is only relevant if there is a separate “informed consent” page or if the survey goes over several pages. This is a measure for attrition. Note that “completion” can involve leaving questionnaire items blank. This is not a measure for how completely questionnaires were filled in. (If you need a measure for this, use the word “completeness rate”.) | see ‘Data Cleaning and Analysis’ and ‘Figure 1’  We included all questionnaires in our analysis, where respondents answered the items of sections (1) to (4). As we did not look into sections (5) and (6) for this paper, a completion rate for the entire questionnaire was not computed. Thus, we measured completion by looking at the first four sections only. |
| **PREVETING MULTIPLE ENTRIES FROM THE SAME INDIVIDUAL** | | | |
| Cookies used | 24 | Indicate whether cookies were used to assign a unique user identifier to each client computer. If so, mention the page on which the cookie was set and read, and how long the cookie was valid. Were duplicate entries avoided by preventing users access to the survey twice; or were duplicate database entries having the same user ID eliminated before analysis? In the latter case, which entries were kept for analysis (e.g., the first entry or the most recent)? | We decided not to use cookies to prevent multiple entries from the same individual, as general practitioners in one practice might share a computer. Thus, we would automatically exclude them from participating in the survey. |
| IP check | 25 | Indicate whether the IP address of the client computer was used to identify potential duplicate entries from the same user. If so, mention the period of time for which no two entries from the same IP address were allowed (e.g., 24 hours). Were duplicate entries avoided by preventing users with the same IP address access to the survey twice; or were duplicate database entries having the same IP address within a given period of time eliminated before analysis? If the latter, which entries were kept for analysis (e.g., the first entry or the most recent)? | see ‘Data Cleaning and Analysis’ and ‘Figure 1’  We checked the anonymized IP addresses of participants to identify duplicate entries from the same individual. However, we only excluded IP address duplicates with identical demographic data to ensure that we would allow general practitioners working in the same practice and thus sharing an IP address to participate in the survey. We kept the most recent and complete entry for the cases with identical IP addresses and demographic data. |
| Log file analysis | 26 | Indicate whether other techniques to analyze the log file for identification of multiple entries were used. If so, please describe. | n/a |
| Registration | 27 | In “closed” (non-open) surveys, users need to login first and it is easier to prevent duplicate entries from the same user. Describe how this was done. For example, was the survey never displayed a second time once the user had filled it in, or was the username stored together with the survey results and later eliminated? If the latter, which entries were kept for analysis (e.g., the first entry or the most recent)? | n/a |
| **ANALYSIS** | | | |
| Handling of incomplete responses | 28 | Were only completed questionnaires analyzed? Were questionnaires which terminated early (where, for example, users did not go through all questionnaire pages) also analyzed? | see ‘Data Cleaning and Analysis’ and ‘Figure 1’  We included all questionnaires in our analysis, where respondents answered the items of sections (1) to (4). As we did not look into sections (5) and (6) for this paper, a completion rate for the entire questionnaire was not computed. Thus, we also analyzed questionnaires that have not provided responses for the last two sections. |
| Questionnaires submitted with atypical timestamp | 29 | Some investigators may measure the time people needed to fill in a questionnaire and exclude questionnaires that were submitted too soon. Specify the timeframe that was used as a cut-off point and describe how this point was determined. | see ‘Data Cleaning and Analysis’ and ‘Figure 1’ |
| Statistical correction | 30 | Indicate whether any methods such as weighting of items or propensity scores have been used to adjust for the non-representative sample; if so, please describe the methods. | n/a |
