# Supplementary material for: Big 5 Personality Traits and Individual- and Practice-Related Characteristics as Influencing Factors of Digital Maturity in General Practices: Quantitative Web-Based Survey Study
Source: J Med Internet Res. 2024 Jan 22;26:e52085. doi: 10.2196/52085 (PMC10845021; doi:10.2196/52085)
Supplement: Multimedia Appendix 5 [file jmir_v26i1e52085_app5.docx]

**Multimedia Appendix 5: Translated survey questionnaire for general practitioners.**

*Disclaimer: The questionnaire was presented to respondents in German. This copy was translated, only stating questions included in the publication.*

***Introduction ________________________________________________________***

Dear Sir or Madam,

Thank you for your interest in this study.

As part of a doctoral thesis at the Faculty of Health at Witten/Herdecke University, we are investigating the degree of digitalization of German general practices as well as your concerns and wishes regarding the adoption of digital health solutions. On this basis, we aim to derive relevant strategies that make it easier for general practitioners and decision-makers to advance the digitalization of the healthcare system.

In this study, we define digital health solutions as digital tools, technologies, and services to improve healthcare, make it more efficient, and personalize it. This includes the use of digital services (e.g., video consultations, digital telephone assistance system, digital appointment booking, digital medical history, digital practice administration) and the use of connected medical devices and artificial intelligence (e.g., telemonitoring, decision support systems).

This survey addresses practicing general practitioners in Germany. The survey will take about 10 to 15 minutes to complete. We would like to ask you to answer the questionnaire completely.

This research project has been approved by the Ethics Committee of Witten/Herdecke University (S-242/2022). On the following pages, you will find all the necessary information on data processing and your rights as a participant. To participate in this study, it is necessary that you electronically provide your informed consent afterward.

Thank you very much!

***Information on data security and storage policies _________________________***

Within this online survey, the personal data of the respondents will be collected and processed anonymously. The data collected is used purely for scientific purposes and cannot be traced to you personally. Due to the anonymous data collection, a subsequent deletion of your data is impossible. Your consent to the data protection regulations is voluntary and can be revoked in writing or verbally at any time without giving reasons and without personal disadvantage.

Show privacy policy.

I have read the information on the survey data processing and agree to participate in the study.

***Questions about yourself and your job __________________________________***

In the following, we would like to learn more about you as a person, your practice, and your interactions with digital health solutions.

By digital health solutions, we mean digital tools, technologies, and services to improve healthcare, make it more efficient, and personalize it. This includes the use of digital services (e.g., video consultations, digital telephone assistance system, digital appointment booking, digital medical history, digital practice administration) and the use of connected medical devices and artificial intelligence (e.g., telemonitoring, decision support systems).

| Which gender do you identify with? | 🞎 man |
| --- | --- |
|  | 🞎 woman |
|  | 🞎 nonbinary |
|  | 🞎 no answer |

| How old are you? | 🞎 younger than 26 years old |
| --- | --- |
|  | 🞎 26 to 35 years old |
|  | 🞎 36 to 45 years old |
|  | 🞎 46 to 55 years old |
|  | 🞎 56 to 65 years old |
|  | 🞎 older than 65 years old |

| Where do you work as a general practitioner?  I work in a village/town with… | 🞎 less than 5,000 inhabitants |
| --- | --- |
|  | 🞎 5,000 to 20,000 inhabitants |
|  | 🞎 20,001 to 100,000 inhabitants |
|  | 🞎 100,001 to 500,000 inhabitants |
|  | 🞎 more than 500,000 inhabitants |
| How many years of professional experience do you currently have? | 🞎 less than 1 year |
|  | 🞎 1 to 5 years |
|  | 🞎 6 to 10 years |
|  | 🞎 11 to 20 years |
|  | 🞎 21 to 30 years |
|  | 🞎 more than 30 years |

| Do you treat patients with statutory and/or private health insurance? | 🞎 only statutory health-insured patients |
| --- | --- |
|  | 🞎 only privately health-insured patients |
|  | 🞎 both |

| In which type of practice do you currently work? | 🞎 single practice |
| --- | --- |
|  | 🞎 practice sharing |
|  | 🞎 group practice |
|  | 🞎 practice clinic |
|  | 🞎 practice network |
|  | 🞎 medical care center |
|  | 🞎 collaborative laboratory |

| How often do you use digital health solutions? | 🞎 never |
| --- | --- |
|  | 🞎 less than once per month |
|  | 🞎 monthly |
|  | 🞎 weekly |
|  | 🞎 daily |

| How likely are you to use digital health solutions in the next 12 months? | 🞎 (1) very unlikely |
| --- | --- |
|  | 🞎 (2) rather unlikely |
|  | 🞎 (3) neither unlikely nor likely |
|  | 🞎 (4) rather likely |
|  | 🞎 (5) very likely |

| How digitally affine do you consider the medical assistants in your practice to be in a professional context? | 🞎 (1) not at all digitally savvy |
| --- | --- |
|  | 🞎 (2) rather not digitally savvy |
|  | 🞎 (3) neither not digitally savvy nor digitally savvy |
|  | 🞎 (4) rather digitally savvy |
|  | 🞎 (5) fully digitally savvy |

***Questions about your interaction with technical systems ___________________***

In the following, we will ask you about your interaction with technical systems. The term ‘technical systems’ refers to apps and other software applications, as well as entire digital devices (e.g., mobile phone, computer, TV, car navigation).

Please indicate the degree to which you agree/disagree with the following statements.

|  | Completely disagree | Largely disagree | Slightly disagree | Slightly agree | Largely agree | Completely agree |
| --- | --- | --- | --- | --- | --- | --- |
| I like to occupy myself in greater detail with technical systems. | 🞎 | 🞎 | 🞎 | 🞎 | 🞎 | 🞎 |
| I like testing the functions of new technical systems. | 🞎 | 🞎 | 🞎 | 🞎 | 🞎 | 🞎 |
| I predominantly deal with technical systems because I have to. | 🞎 | 🞎 | 🞎 | 🞎 | 🞎 | 🞎 |
| When I have a new technical system in front of me, I try it out intensively. | 🞎 | 🞎 | 🞎 | 🞎 | 🞎 | 🞎 |
| I enjoy spending time becoming acquainted with a new technical system. | 🞎 | 🞎 | 🞎 | 🞎 | 🞎 | 🞎 |
| It is enough for me that a technical system works; I don’t care how or why. | 🞎 | 🞎 | 🞎 | 🞎 | 🞎 | 🞎 |
| I try to understand how a technical system exactly works. | 🞎 | 🞎 | 🞎 | 🞎 | 🞎 | 🞎 |
| It is enough for me to know the basic functions of a technical system. | 🞎 | 🞎 | 🞎 | 🞎 | 🞎 | 🞎 |
| I try to make full use of the capabilities of a technical system. | 🞎 | 🞎 | 🞎 | 🞎 | 🞎 | 🞎 |

***Questions about your personality ______________________________________***

In the following, we would like to learn more about you as a person. For each of the following statements, please indicate how much it applies to you. You can grade your answers from "does not apply at all" to "fully applies".

|  | Does not apply at all | Does rather not apply | Neither not applies nor applies | Rather applies | Fully applies |
| --- | --- | --- | --- | --- | --- |
| I am rather shy, reserved. | 🞎 | 🞎 | 🞎 | 🞎 | 🞎 |
| I tend to criticize others. | 🞎 | 🞎 | 🞎 | 🞎 | 🞎 |
| I do tasks thoroughly. | 🞎 | 🞎 | 🞎 | 🞎 | 🞎 |
| I become depressed easily. | 🞎 | 🞎 | 🞎 | 🞎 | 🞎 |
| I have a wide range of interests. | 🞎 | 🞎 | 🞎 | 🞎 | 🞎 |
| I am enthusiastic and can easily excite others. | 🞎 | 🞎 | 🞎 | 🞎 | 🞎 |
| I trust others easily and believe in the good in people. | 🞎 | 🞎 | 🞎 | 🞎 | 🞎 |
| I am comfortable and tend to be lazy. | 🞎 | 🞎 | 🞎 | 🞎 | 🞎 |
| I am relaxed and don’t let stress upset me. | 🞎 | 🞎 | 🞎 | 🞎 | 🞎 |
| I am thoughtful and like to think about things. | 🞎 | 🞎 | 🞎 | 🞎 | 🞎 |
| I am rather the ‘quiet type’ and taciturn. | 🞎 | 🞎 | 🞎 | 🞎 | 🞎 |
| I can be cold and distant. | 🞎 | 🞎 | 🞎 | 🞎 | 🞎 |
| I am efficient and work quickly. | 🞎 | 🞎 | 🞎 | 🞎 | 🞎 |
| I worry a lot. | 🞎 | 🞎 | 🞎 | 🞎 | 🞎 |
| I have an active imagination and am creative. | 🞎 | 🞎 | 🞎 | 🞎 | 🞎 |
| I am outgoing and sociable. | 🞎 | 🞎 | 🞎 | 🞎 | 🞎 |
| I can be brusque and dismissive towards others. | 🞎 | 🞎 | 🞎 | 🞎 | 🞎 |
| I make plans and execute them. | 🞎 | 🞎 | 🞎 | 🞎 | 🞎 |
| I become nervous and insecure easily. | 🞎 | 🞎 | 🞎 | 🞎 | 🞎 |
| I appreciate artistic and aesthetic impressions. | 🞎 | 🞎 | 🞎 | 🞎 | 🞎 |
| I have little artistic interest. | 🞎 | 🞎 | 🞎 | 🞎 | 🞎 |

***Questions about the digital maturity of your practice (part 1) ________________***

In the following, we would like to know more about your practice. For each of the following statements, please indicate how much you agree with them. You can grade your answers from “strongly disagree” to “strongly agree”.

|  | Strongly disagree | Rather disagree | Neither disagree nor agree | Rather agree | Strongly agree |
| --- | --- | --- | --- | --- | --- |
| In my practice, every team member is supported in the implementation of digital tools. | 🞎 | 🞎 | 🞎 | 🞎 | 🞎 |
| My practice handles data and information responsibly and confidentially. | 🞎 | 🞎 | 🞎 | 🞎 | 🞎 |
| I guide and lead team members as they implement new digital tools. | 🞎 | 🞎 | 🞎 | 🞎 | 🞎 |
| In my practice, risks are actively identified and tracked to ensure information security. | 🞎 | 🞎 | 🞎 | 🞎 | 🞎 |
| In my practice, standards and structures have been agreed upon and established. | 🞎 | 🞎 | 🞎 | 🞎 | 🞎 |
| Our practice culture is participatory and inclusive and values innovation. | 🞎 | 🞎 | 🞎 | 🞎 | 🞎 |
| My practice hast he right hardware and network resources to use digital tools. | 🞎 | 🞎 | 🞎 | 🞎 | 🞎 |
| The digital tools in my practice are of good quality. | 🞎 | 🞎 | 🞎 | 🞎 | 🞎 |
| My practice offers video consultations. | 🞎 | 🞎 | 🞎 | 🞎 | 🞎 |
| In my practice, patients can book, edit, and cancel appointments digitally. | 🞎 | 🞎 | 🞎 | 🞎 | 🞎 |
| My practice uses an automated, digital telephone assistance system. | 🞎 | 🞎 | 🞎 | 🞎 | 🞎 |
| My practice regularly uses e-prescriptions. | 🞎 | 🞎 | 🞎 | 🞎 | 🞎 |
| My practice allows new patients to complete their medical history digitally. | 🞎 | 🞎 | 🞎 | 🞎 | 🞎 |
| In my practice, billing and financial management are completely digital. | 🞎 | 🞎 | 🞎 | 🞎 | 🞎 |
| My practice schedules, organizes, and records shifts and work hours digitally. | 🞎 | 🞎 | 🞎 | 🞎 | 🞎 |

***Questions about the digital maturity of your practice (part 2) ________________***

In the following, we would like to know more about your practice. For each of the following statements, please indicate how much you agree with them. You can grade your answers from “strongly disagree” to “strongly agree”.

|  | Strongly disagree | Rather disagree | Neither disagree nor agree | Rather agree | Strongly agree |
| --- | --- | --- | --- | --- | --- |
| My practice actively aims to provide ongoing training and education to team members. | 🞎 | 🞎 | 🞎 | 🞎 | 🞎 |
| In my practice, team members actively share knowledge with each other. | 🞎 | 🞎 | 🞎 | 🞎 | 🞎 |
| I use digital systems as intended. | 🞎 | 🞎 | 🞎 | 🞎 | 🞎 |
| The team members in my practice use digital systems as intended. | 🞎 | 🞎 | 🞎 | 🞎 | 🞎 |
| My practice digitally exchanges information with hospitals or other external partners. | 🞎 | 🞎 | 🞎 | 🞎 | 🞎 |
| The digital systems within my practice are interconnected and exchange information. | 🞎 | 🞎 | 🞎 | 🞎 | 🞎 |
| My practice operates flexibly and is able to adapt to internal or external changes. | 🞎 | 🞎 | 🞎 | 🞎 | 🞎 |
| MY practice has a clear digital agenda that aligns with medical and business objectives. | 🞎 | 🞎 | 🞎 | 🞎 | 🞎 |
| My practice analyzes and leverages existing patient data for effective decision making. | 🞎 | 🞎 | 🞎 | 🞎 | 🞎 |
| My practice uses medical data to identify necessary adjustments in patient care early on. | 🞎 | 🞎 | 🞎 | 🞎 | 🞎 |
| Our use of digital tools encourages patients to actively participate in health decisions. | 🞎 | 🞎 | 🞎 | 🞎 | 🞎 |
| My practice involves patients in decisions about the implementation of digital tools. | 🞎 | 🞎 | 🞎 | 🞎 | 🞎 |
| My practice shares data digitally with patients so they can view, record, and edit them. | 🞎 | 🞎 | 🞎 | 🞎 | 🞎 |

***Questions about perceived barriers _____________________________________***

*Not presented here as this section is part of subsequent analysis and will be published elsewhere.*

***Questions about potential measures to support adoption __________________***

*Not presented here as this section is part of subsequent analysis and will be published elsewhere.*

***Conclusion and goodbye _____________________________________________***

You have reached the end of this survey. Your responses have now been recorded.

Thank you very much for your participation!

Do you have colleagues who might also be interested in participating? If so, please forward the survey link to them: survey link.

You may now close the browser window.
