# Supplementary material for: Big 5 Personality Traits and Individual- and Practice-Related Characteristics as Influencing Factors of Digital Maturity in General Practices: Quantitative Web-Based Survey Study
Source: J Med Internet Res. 2024 Jan 22;26:e52085. doi: 10.2196/52085 (PMC10845021; doi:10.2196/52085)
Supplement: Multimedia Appendix 6 [file jmir_v26i1e52085_app6.docx]

**Multimedia Appendix 6: Overview of regression coefficients for the regression models predicting digital maturity (N = 219).**

| **Model** | **Variable** | **b** | **SE B** | ***β*** | ***P* value** | **95% CI** |
| --- | --- | --- | --- | --- | --- | --- |
| **1** | **(Constant)** | 3.429 | 0.262 | — | <.001 | 2.91 ; 3.94 |
|  | **Gender: women versus men** | −0.173 | 0.094 | −.135 | .07 | −0.35 ; 0.01 |
|  | **Age: 36-45 versus 26-35** | 0.035 | 0.184 | .025 | .85 | −0.32 ; 0.39 |
|  | **Age: 46-55 versus 26-35** | 0.068 | 0.220 | .047 | .76 | −0.36 ; 0.50 |
|  | **Age: 56-65 versus 26-35** | −0.063 | 0.263 | −.047 | .81 | −0.58 ; 0.45 |
|  | **Age: >65 versus 26-35** | −0.066 | 0.348 | −.022 | .85 | −0.75 ; 0.61 |
|  | **Practice location size: 5,000-20,000 versus <5,000** | −0.111 | 0.134 | −.080 | .41 | −0.37 ; 0.15 |
|  | **Practice location size: 20,001-100,000 versus <5,000** | −0.078 | 0.141 | −.049 | .58 | −0.35 ; 0.20 |
|  | **Practice location size: 100,001-500,000 versus <5,000** | −0.063 | 0.160 | −.033 | .69 | −0.37 ; 0.25 |
|  | **Practice location size: >500,000 versus <5,000** | −0.103 | 0.147 | −.059 | .49 | −0.39 ; 0.18 |
|  | **Professional experience: 6-10 versus 1-5** | 0.106 | 0.183 | .061 | .56 | −0.25 ; 0.46 |
|  | **Professional experience: 11-20 versus 1-5** | 0.251 | 0.188 | .175 | .18 | −0.11 ; 0.62 |
|  | **Professional experience: 21-30 versus 1-5** | 0.288 | 0.233 | .202 | .22 | −0.17 ; 0.74 |
|  | **Professional experience: >30 versus 1-5** | 0.423 | 0.266 | .268 | .11 | −0.10 ; 0.94 |
|  | **Practice type: Practice sharing versus single practice** | 0.060 | 0.204 | .021 | .77 | −0.34 ; 0.46 |
|  | **Practice type: Group practice versus single practice** | −0.061 | 0.098 | −.048 | .54 | −0.25 ; 0.13 |
|  | **Practice type: Medical care center versus single practice** | −0.109 | 0.209 | −.039 | .60 | −0.52 ; 0.30 |
| **2** | **(Constant)** | 2.903 | 0.331 | — | <.001 | 2.25 ; 3.55 |
|  | **Gender: women versus men** | −0.132 | 0.096 | −.103 | .17 | -0.32 ; 0.05 |
|  | **Age: 36-45 versus 26-35** | 0.036 | 0.181 | .025 | .84 | −0.32 ; 0.39 |
|  | **Age: 46-55 versus 26-35** | 0.099 | 0.217 | .067 | .65 | −0.32 ; 0.52 |
|  | **Age: 56-65 versus 26-35** | 0.029 | 0.261 | .022 | .91 | −0.48 ; 0.54 |
|  | **Age: >65 versus 26-35** | 0.146 | 0.350 | .048 | .68 | −0.54 ; 0.83 |
|  | **Practice location size: 5,000-20,000 versus <5,000** | −0.098 | 0.132 | −.071 | .46 | −0.35 ; 0.16 |
|  | **Practice location size: 20,001-100,000 versus <5,000** | −0.120 | 0.140 | −.076 | .39 | −0.39 ; 0.15 |
|  | **Practice location size: 100,001-500,000 versus <5,000** | −0.079 | 0.158 | −.041 | .62 | −0.39 ; 0.23 |
|  | **Practice location size: >500,000 versus <5,000** | −0.092 | 0.145 | −.053 | .53 | −0.37 ; 0.19 |
|  | **Professional experience: 6-10 versus 1-5** | 0.083 | 0.183 | .048 | .65 | −0.27 ; 0.44 |
|  | **Professional experience: 11-20 versus 1-5** | 0.217 | 0.187 | .151 | .25 | −0.15 ; 0.58 |
|  | **Professional experience: 21-30 versus 1-5** | 0.241 | 0.231 | .169 | .30 | −0.21 ; 0.69 |
|  | **Professional experience: >30 versus 1-5** | 0.308 | 0.266 | .196 | .25 | −0.21 ; 0.83 |
|  | **Practice type: Practice sharing versus single practice** | 0.007 | 0.202 | .003 | .97 | −0.39 ; 0.40 |
|  | **Practice type: Group practice versus single practice** | −0.105 | 0.098 | −.082 | .28 | −0.29 ; 0.08 |
|  | **Practice type: Medical care center versus single practice** | −0.143 | 0.207 | −.051 | .49 | −0.55 ; 0.26 |
|  | **Current usage: Less than once per month versus never** | 0.011 | 0.183 | .007 | .95 | −0.35 ; 0.37 |
|  | **Current usage: Monthly versus never** | −0.088 | 0.224 | −.043 | .69 | −0.52 ; 0.35 |
|  | **Current usage: Weekly versus never** | −0.195 | 0.222 | −.108 | .38 | −0.63 ; 0.24 |
|  | **Current usage: Daily versus never** | −0.064 | 0.223 | −.050 | .78 | −0.50 ; 0.37 |
|  | **Future usage** | 0.137 | 0.056 | .269 | .02 | 0.02 ; 0.24 |
| **3** | **(Constant)** | 1.702 | 0.378 | — | <.001 | 0.95 ; 2.44 |
|  | **Gender: women versus men** | −0.012 | 0.093 | −.009 | .90 | -0.19 ; 0.17 |
|  | **Age: 36-45 versus 26-35** | −0.013 | 0.169 | −.009 | .94 | −0.34 ; 0.32 |
|  | **Age: 46-55 versus 26-35** | 0.116 | 0.202 | .079 | .57 | −0.28 ; 0.51 |
|  | **Age: 56-65 versus 26-35** | 0.062 | 0.244 | .046 | .80 | −0.41 ; 0.54 |
|  | **Age: >65 versus 26-35** | 0.134 | 0.327 | .044 | .68 | −0.51 ; 0.77 |
|  | **Practice location size: 5,000-20,000 versus <5,000** | −0.182 | 0.125 | −.132 | .15 | −0.42 ; 0.06 |
|  | **Practice location size: 20,001-100,000 versus <5,000** | −0.081 | 0.132 | −.051 | .54 | −0.34 ; 0.17 |
|  | **Practice location size: 100,001-500,000 versus <5,000** | −0.077 | 0.148 | −.040 | .60 | −0.36 ; 0.21 |
|  | **Practice location size: >500,000 versus <5,000** | −0.092 | 0.136 | −.053 | .50 | −0.35 ; 0.17 |
|  | **Professional experience: 6-10 versus 1-5** | 0.185 | 0.172 | .106 | .28 | −0.15 ; 0.52 |
|  | **Professional experience: 11-20 versus 1-5** | 0.287 | 0.175 | .200 | .10 | −0.05 ; 0.63 |
|  | **Professional experience: 21-30 versus 1-5** | 0.274 | 0.216 | .192 | .21 | −0.15 ; 0.69 |
|  | **Professional experience: >30 versus 1-5** | 0.305 | 0.249 | .194 | .22 | −0.18 ; 0.79 |
|  | **Practice type: Practice sharing versus single practice** | 0.005 | 0.189 | .002 | .98 | −0.36 ; 0.37 |
|  | **Practice type: Group practice versus single practice** | −0.074 | 0.091 | −.057 | .42 | −0.25 ; 0.10 |
|  | **Practice type: Medical care center versus single practice** | −0.189 | 0.194 | −.068 | .33 | −0.57 ; 0.19 |
|  | **Current usage: Less than once per month versus never** | 0.063 | 0.172 | .040 | .71 | −0.27 ; 0.40 |
|  | **Current usage: Monthly versus never** | −0.038 | 0.209 | −.019 | .86 | −0.45 ; 0.37 |
|  | **Current usage: Weekly versus never** | −0.159 | 0.207 | −.088 | .44 | −0.56 ; 0.24 |
|  | **Current usage: Daily versus never** | −0.083 | 0.208 | −.065 | .69 | −0.49 ; 0.32 |
|  | **Future usage** | 0.116 | 0.052 | .228 | .03 | 0.01 ; 0.21 |
|  | **Perceived digital affinity of medical assistants** | 0.153 | 0.044 | .234 | .001 | 0.06 ; 0.23 |
|  | **GPs’ digital affinity** | 0.144 | 0.041 | .247 | .001 | 0.06 ; 0.22 |
| **4** | **(Constant)** | 1.702 | 0.515 | — | .001 | 0.68 ; 2.71 |
|  | **Gender: women versus men** | 0.022 | 0.096 | .017 | .82 | −0.16 ; 0.21 |
|  | **Age: 36-45 versus 26-35** | 0.140 | 0.169 | .098 | .41 | −0.19 ; 0.47 |
|  | **Age: 46-55 versus 26-35** | 0.281 | 0.201 | .192 | .16 | −0.11 ; 0.67 |
|  | **Age: 56-65 versus 26-35** | 0.217 | 0.242 | .161 | .37 | −0.26 ; 0.69 |
|  | **Age: >65 versus 26-35** | 0.290 | 0.320 | .095 | .37 | −0.34 ; 0.92 |
|  | **Practice location size: 5,000-20,000 versus <5,000** | −0.160 | 0.124 | −.116 | .20 | −0.40 ; 0.08 |
|  | **Practice location size: 20,001-100,000 versus <5,000** | −0.071 | 0.129 | −.045 | .58 | −0.32 ; 0.18 |
|  | **Practice location size: 100,001-500,000 versus <5,000** | −0.075 | 0.144 | −.039 | .60 | −0.35 ; 0.20 |
|  | **Practice location size: >500,000 versus <5,000** | −0.108 | 0.138 | −.062 | .43 | −0.38 ; 0.16 |
|  | **Professional experience: 6-10 versus 1-5** | 0.202 | 0.168 | .117 | .23 | −0.12 ; 0.53 |
|  | **Professional experience: 11-20 versus 1-5** | 0.205 | 0.172 | .143 | .23 | −0.13 ; 0.54 |
|  | **Professional experience: 21-30 versus 1-5** | 0.187 | 0.213 | .131 | .38 | −0.23 ; 0.60 |
|  | **Professional experience: >30 versus 1-5** | 0.192 | 0.245 | .122 | .44 | −0.29 ; 0.67 |
|  | **Practice type: Practice sharing versus single practice** | 0.009 | 0.184 | .003 | .96 | −0.35 ; 0.37 |
|  | **Practice type: Group practice versus single practice** | −0.051 | 0.089 | −.039 | .57 | −0.22 ; 0.12 |
|  | **Practice type: Medical care center versus single practice** | −0.136 | 0.189 | −.049 | .47 | −0.50 ; 0.23 |
|  | **Current usage: Less than once per month versus never** | 0.147 | 0.169 | .094 | .39 | −0.18 ; 0.48 |
|  | **Current usage: Monthly versus never** | 0.113 | 0.206 | .055 | .58 | −0.29 ; 0.51 |
|  | **Current usage: Weekly versus never** | −0.055 | 0.204 | −.030 | .79 | −0.45 ; 0.34 |
|  | **Current usage: Daily versus never** | −0.034 | 0.205 | −.026 | .87 | −0.43 ; 0.37 |
|  | **Future usage** | 0.105 | 0.051 | .205 | .04 | 0.00 ; 0.20 |
|  | **Perceived digital affinity of medical assistants** | 0.147 | 0.044 | .226 | .001 | 0.06 ; 0.23 |
|  | **GPs’ digital affinity** | 0.145 | 0.041 | .250 | <.001 | 0.06 ; 0.22 |
|  | **Extraversion** | 0.034 | 0.058 | .043 | .56 | −0.08 ; 0.14 |
|  | **Agreeableness** | −0.007 | 0.055 | −.009 | .89 | −0.11 ; 0.10 |
|  | **Conscientiousness** | 0.121 | 0.078 | .110 | .12 | −0.03 ; 0.27 |
|  | **Neuroticism** | −0.216 | 0.065 | −.238 | .001 | −0.34 ; −0.08 |
|  | **Openness** | −0.060 | 0.062 | −.065 | .33 | -0.18 ; 0.06 |

**Note:** Table shows the regression coefficients for our regression model. SE = standard error for regression coefficient b; 95% CI presents upper and lower bound for the 95% confidence interval.
